# Phylogenetic and Morphological Perspectives on *Crepidotus* subg. *Dochmiopus*: Exploratively Unveiling Hidden Diversity in China

**DOI:** 10.3390/jof10100710

**Published:** 2024-10-11

**Authors:** Menghui Han, Qin Na, Renxiu Wei, Hui Zeng, Yaping Hu, Libo Zhang, Jinhong Du, Li Zou, Weimin Tang, Xianhao Cheng, Yupeng Ge

**Affiliations:** 1 Institute of Mycological Science and Technology, School of Horticulture, Ludong University, Yantai 264025, China; han_mh8128@163.com (M.H.); naqin19890317@163.com (Q.N.); wrx_1260@163.com (R.W.); chengxianhao@sohu.com (X.C.); 2 Institute of Edible Fungi, Fujian Academy of Agricultural Sciences, Fuzhou 350014, China; zenghui@faas.cn; 3 National and Local Joint Engineering Research Center for Breeding & Cultivation of Features Edible Fungi, Fuzhou 350014, China; 4 Nanjing Institute of Environmental Sciences, Ministry of Ecology and Environment, State Environmental Protection Scientific Observation and Research Station for Ecological Environment of Wuyi Mountains, Nanjing 210042, China; huyap9009@163.com; 5 Institute of Ecology, Chinese Research Academy of Environmental Science, Beijing 100012, China; zhang.libo@craes.org.cn (L.Z.); dujh@craes.org.cn (J.D.); 6 College of Forestry, Northeast Forestry University, Harbin 150040, China; shyj@nefu.edu.cn; 7 Wufeng Tujia Autonomous County Environmental Monitoring Station, Yichang 443400, China; booktwm@163.com

**Keywords:** new taxa, taxonomy, systematics, dark-spored agarics, saprophytic fungi

## Abstract

*Crepidotus* subg. *Dochmiopus* contributes to more than half of *Crepidotus* species and exhibits highly hidden diversity. However, *C*. subg. *Dochmiopus* is challenging to study because the basidiomata of *C*. subg. *Dochmiopus* species are usually small and white, inconspicuous interspecific distinctions, and possess a familiar complex. In this study, we utilized a variety of characteristics for species identification, including habitat, presence or absence of a stipe in mature specimens, pileipellis and cheilocystidia patterns, whether the lamellae edges are fimbriated, and other characteristics. Above all, cheilocystidia and pileipellis patterns will be important in *C*. subg. *Dochmiopus* research. Based on the present specimens, we constructed a multigene phylogenetic tree (ITS + LSU) and recognized four new species: *C. lamellomaculatus* sp. nov., *C. capitatocystidiatus* sp. nov., *C. succineus* sp. nov., *C. clavocystidiatustustus* sp. nov. Detailed morphological descriptions, photographs, line drawings and comparisons with closely related taxa for the new species are provided. The current phylogenetic analysis does not support the previously classifications, indicating that the classification of *Crepidotus* requires re-evaluation. But the existing molecular datasets and species’ descriptions are insufficient to fully resolve the classification. Further integration of new gene segments and a comprehensive review of morphological characteristics will reveal a natural classification for *Crepidotus*.

## 1. Introduction

*Crepidotus* (Fr.) Staude are notable saprophytic fungi, primarily utilizing decaying wood and branches as growth substrates, characterized by small basidiomata with dark-colored basidiospores, and these mostly exhibit an underdeveloped stipe [1,2,3,4]. Since Staude elevated the *Agaricus* L. tribe *Crepidotus* Fr. to generic rank in 1857, a total of 586 *Crepidotus* taxa have been recorded, encompassing approximately 400 species (https://www.indexfungorum.org/Names/Names.asp, accessed on 5 October 2024). Taxonomic research about this genus has traditionally been concentrated in Europe and America [1,2,3,5,6]. Since the early 21st century, studies in Asia have gradually increased, and 34 species have been published, with 13 of these species described by Chinese scholars [7,8,9,10,11,12,13,14,15]. In 1965, based on morphological studies, Hesler and Smith classified *Crepidotus* into three subgenera [5], and documented 123 taxa: 19 clampless taxa in *C.* subg. *Crepidotus* Hesler & A.H. Sm, 61 elongated-spored taxa in *C.* subg. *Dochmiopus* (Pat.) Pilát, 43 globose-spored taxa in *C.* subg. *Sphaerula* Hesler & A.H. Sm. In 2008, Consiglio and Setti [6], primarily focused on European specimens, and revised the classification by dividing the genus into two subgenera and combining globose-spored species into *C.* subg. *Dochmiopus* as sect. *Sphaeruli* Hesler & A.H. Sm. They documented including 25 taxa in the genus *Crepidotus*: 6 in *C.* subg. *Crepidotus* and 19 in *C.* subg. *Dochmiopus* 12 elongated-spored and 7 globose-spored taxa [6]. Both classifications indicate that *C.* subg. *Dochmiopus* exhibited abundant species diversity, representing about 50% of taxa of the genus [5,6]. Despite different definitions of *C.* subg. *Dochmiopus*, most taxa within this subgenus are characterized by ornamented basidiospores and hyphae with clamp connections [1,5,6].

*Crepidotus* subg. *Dochmiopus* exhibits simple characteristics, limited taxonomic characteristics, insufficiently detailed morphological descriptions, and absent available molecular sequences, presenting significant challenges for precise identification and systematic classification. Initially described by Pilát in 1948, *C.* subg. *Dochmiopus* is characterized by its small and white basidiomata, non-hygrophanous, and fibrillose-covered pileus [1,5,16]. The macroscopic characteristics within this subgenus are highly similar, and only basidiospores, cystidia and pileipellis demonstrate morphological value. The restriction of utilized morphological characteristics brought obstacles to the efficiency of identification and classification. The majority of species within *C.* subg. *Dochmiopus* present cheilocystidia while lacking pleurocystidia or pileocystidia, only with a few exceptions. For instance, four species within *C.* subg. *Dochmiopus* sect. *Cystiodiosi* Hesler & A.H. Sm (*C. pseudoflammeus* Hesler & A.H. Sm., *C. albatus* Hesler & A.H. Sm., *C. luteicolor* Hesler & A.H. Sm., *C. rainierensis* Hesler & A.H. Sm.) possess pleurocystidia [5]. In addition, *C. autochthonus* J. Lang, *C. herrerae* Bandala & Montoya, *C. malachioides* Consiglio, Prydiuk & Setti, and others possess pileocystidia [6,17]. Given the high species diversity within the genus *Crepidotus,* the scarcity of valuable morphological characteristics further complicates identification and classification. Additionally, some species exhibit variability in cheilocystidia shape, which obscures determinations between inter- and intraspecific variations [18]. For example, *C. variabilis* P. Kummer, the type species of *C.* subg. *Dochmiopus*, possesses six varieties and exhibits diverse morphologies in its cheilocystidia [18]. Jančovičová studied the holotype of *C. variabilis* and noted that the cheilocystidia were predominantly narrowly lageniform or utriform to lobate, with a few being clavate to lageniform with forked and antler-like shapes [18]. Factors such as the maturing status of basidiomata and environmental humidity may lead to the secondary development of cheilocystidia, resulting in substantial intraspecific morphological variability [19]. Such phenomena complicate the accurate determination of the mature morphology of cheilocystidia. Furthermore, accurate recognition is further encumbered by subtle distinctions among closely related species in basidiospore ornamentations, which may vary from smooth to punctate or reticulate patterns [1,6,7]. Presently, GenBank houses fewer than 600 *Crepidotus* sequences, with about 65% being ITS sequences and 35% LSU sequences. These cover 96 identified species and 70 uncertain taxa (https://www.ncbi.nlm.nih.gov/genbank/, accessed on 5 October 2024). Some sequences are less reliable, due to incorrect submissions, thereby reducing their utility. Only around 40 taxa within *C.* subg. *Dochmiopus* have associated sequences, providing limited data for taxonomic and phylogenetic study. Some species were published based on traditional morphological studies and lacked sequences, which may lead to nomenclatural synonyms and cause more difficulties for future research.

The species diversity in this genus has been consistently underestimated. In recent years, our research group has published 12 new species of *Crepidotus* from China [4,7,12,15,20]. However, our ongoing studies of collected specimens have revealed more unknown species. Among these, the number of *C.* subg. *Dochmiopus* is the highest, indicating significant hidden diversity. The simple morphological characteristics, the scarcity of useful taxonomic characteristics, and the limited molecular datasets pose challenges for species recognition and classification. Current phylogenetic analyses do not support the morphologically established classification, and the absence of clamp connections and smooth basidiospores is no longer an exclusive characteristic of *C.* subg. *Crepidotus.* In this study, we introduce four new species of *C.* subg. *Dochmiopus* collected from various regions in China, provide a preliminary discussion of their key taxonomic characteristics and build a multigene phylogenetic tree to discuss taxonomic problems. We consider *C.* subg. *Dochmiopus* contains a hidden species diversity, relying solely on morphological characteristics to distinguish subgenera is not suitable, and there may be new sections within *C.* subg. *Dochmiopus.* More molecular datasets and key taxonomic characteristics could potentially offer robust support to resolve *C.* subg. *Dochmiopus’s* taxonomic problems and establish a more natural classification.

## 2. Materials and Methods

### 2.1. Specimens and Morphological Examination

The studied specimens were collected from Heilongjiang Province, Jilin Province, Shandong Province, Hubei Province, and Fujian Province of China. Macro-morphological descriptions relied on field records of fresh specimens, while micro-morphological descriptions were based on observations of dry specimens. During fieldwork, high-resolution photographs of fresh basidiomata and their surrounding habitat were taken by an Olympus E-M1 Mark III camera equipped with an M. Zuiko Digital Ed 12–40 mm or 60 mm lens (Olympus, Tokyo, Japan) and a Sony ILCE-A7M3 (Sony, Tokyo, Japan) with a Sigma 70 mm f2.8 EX DG MACRO (Sigma, Kobe, Japan). Comprehensive details of the specimens were recorded, including the size, color, shape, and characteristics of pileus, lamellae, stipe, odor and taste of fresh specimens, and their location information. More characters of pileus and lamellae were recorded, including whether the pileus surface was hygrophanous, striate, smooth, villose, or scaly, and whether the lamellae edge was fimbriated. In our descriptions, the color codes and notations for the macroscopic characteristics of the basidiomata followed the work of Robert Ridgway [21]. Freshly collected specimens were dried with portable dryers at 40 °C (Stöckli, Netstal), and then sealed and stored with allochroic silica gel [22]. Microscopic examinations were carried out on dried materials rehydrated with 5% potassium hydroxide (KOH) aqueous solution and, if necessary, stained with 1% Congo red aqueous solution [23,24]. Each microstructure was observed using a Lab A1 microscope (Carl Zeiss AG, Jena, Germany), and then photographed and measured with ZEN 2.3 software [25].At least 20 matured basidiospores of each basidiomata were measured in lateral view (under oil, 1000×), and one or two basidiomata were recorded for each specimen. The notation [a/b/c] used at the beginning of each basidiospore description indicates that a number of basidiospores from b number of basidiomata of c number of specimens were measured; the dimensions (length × width) are presented as (d)e–f–g(h) × (i)j–k–l(m), in which d is the minimum length, e–g represents the range of at least 90% of values, f is the average length and h is the maximum length; the width (i–m) is expressed in the same way [23,26]. Furthermore, Q denotes the ratio of length and width, and Q_m_ refers to the average Q value of all basidiospores ± the sample standard deviation [24,27,28]. The type of basidiospore ornamentation referred to Consiglio & Setti’s descriptions [6]. The length and width of the hyphae of pileipellis, basidia, cheilocystidia, and pileocystidia were measured through at least 20 measurements of each species; for basidia and cystidia, the widest point value was chosen as the width, and the length of the sterigma was excluded from the measurement and measured separately. Based on the observation of the macro- and microstructures of multiple specimens, illustrations of the basidiomata and microstructures of the new species were created. First, pencil sketches were drawn on A4 paper, and then transposed to tracing paper using a needle pen. Subsequently, the line drawings on the tracing paper were digitized into TIFF format using a Canon LiDE120 scanner (Canon, Tokyo, Japan), and post-processed using Photoshop 2020. The voucher specimens used were deposited in the Fungarium of the Fujian Academy of Agricultural Sciences (FFAAS), Fuzhou, China.

### 2.2. DNA Extraction, PCR Amplification and DNA Sequencing

Genomic DNA was extracted from each specimen using the NuClean Plant Genomic DNA Kit (Kangwei Century Biotechnology Co., Beijing, China). The nuclear internal transcribed spacer (ITS) region (ITS1-5.8s-ITS2) and the large subunit region (LSU) of rDNA were amplified by primer pair ITS1/ITS4 and LROR/LR7 [29,30]. Amplifications were performed in a total volume of 25 μL, containing 1 μL of each primer, 12.5 μL 2 × Utaq PCR MasterMix (ZomanBio, Beijing, China), 8.5 μL ddH_2_O, and 2 μL DNA template [31]. The PCR thermocycling protocol for amplification of the ITS region was 94 °C for 4 min, followed by 34 cycles of 94 °C for 45 s, 52 °C for 45 s, and 72 °C for 1 min and final extension for 10 min at 72 °C; for LSU, it was 95 °C for 5 min, followed by 30 cycles of 95 °C for 30 s, 55 °C for 30 s, 72 °C for 1 min and final extension for 10 min at 72 °C [23]. The PCR product was sequenced by Bgi Tech Solutions (Beijing Liuhe) Co. (Beijing, China). The BioEdit v7.0.4.1 was used to inspect chromatograms to ensure the new sequence quality [32,33]. The low-quality sequences were enhanced using plasmid vectors by using VELUTE Gel Mini Purification and pBLUE-T kits (Beijing Zoman Biotechnology Co., Beijing, China) to improve [4]. All obtained sequences in this study were submitted to GenBank. The BLAST and the GenBank (https://www.ncbi.nlm.nih.gov/genbank/, accessed on 5 October 2024) were used to download homologous ITS and LSU sequences of *Crepidotus* and outgroup taxa sequences. Representative species that share a morphological or phylogenetic affinity with the new species were selected for phylogenetic analysis. Detailed information on these specimens is given in Table 1. 

### 2.3. Sequence Alignment and Molecular Phylogeny

This study is based on the use of combined ITS and LSU analyses to investigate the relationship of the new taxa with other species of *Crepidotus*. Maximum likelihood (ML) and Bayesian inferences (BI) were implemented to construct phylogenetic trees. Sequences used for phylogenetic analysis were aligned using MAFFT v7.487, strategy Auto (FFT-NS-1, FFT-NS-2, FFT-NS-i or L-INS-i), and then manually adjusted and trimmed using BioEdit v7.0.4.1. MEGA 10 was used to generate matrixes [32,33,54,55]. The raxmlGUI v2.0 inferred the best-fit substitution model for each gene partition and performed the ML analysis with 1000 bootstrap replicates [56]. For BI analysis with MrBayes v3.2.6, the best-fit substitution model for each gene partition was inferred by MrModeltest 2.3 [57,58]. MCMC generations were run 8 chains for 5,000,000 generations, sampled every 1000 generations, until the average standard deviation of split frequencies was under 0.01. In total, 25% of the trees were removed as a burn-in phase of each analysis [57]. Tracer v1.7.2 was used for the visualization and examination of MCMC trace files from Bayesian phylogenetic inference [59]. Phylogenetic trees were visualized and checked in Figtree v1.4.3 and Photoshop 2020 was used for the final editing of the trees.

## 3. Results

### 3.1. Phylogenetic Analysis

**Figure 1:**
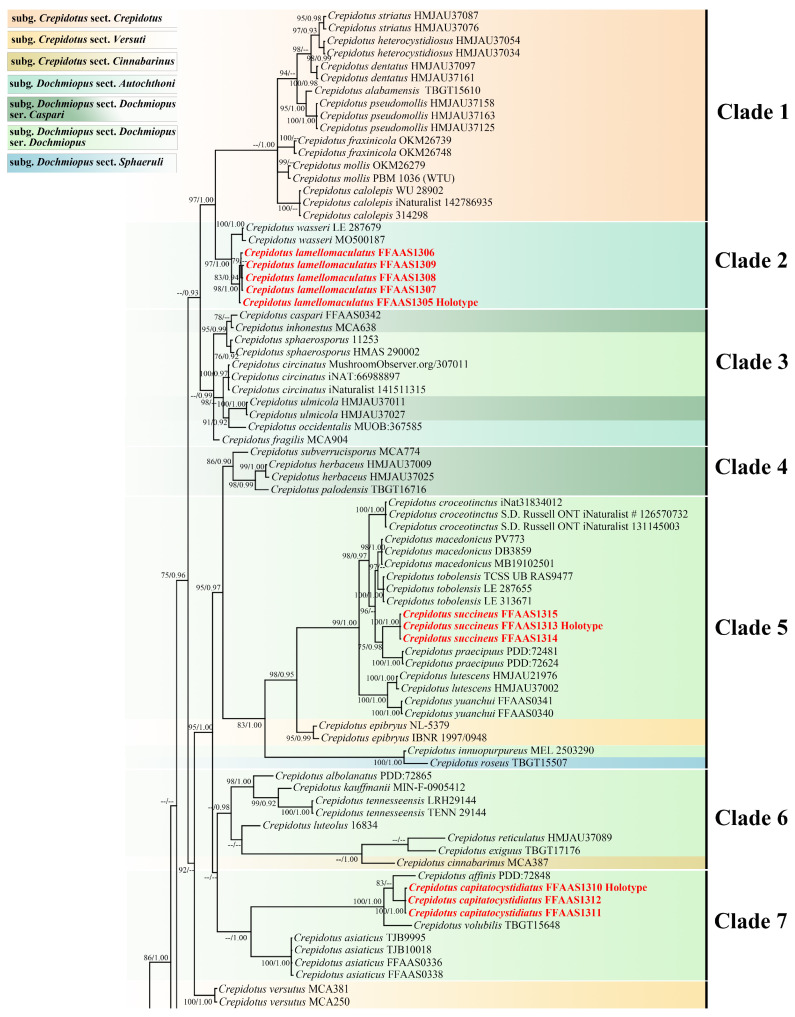

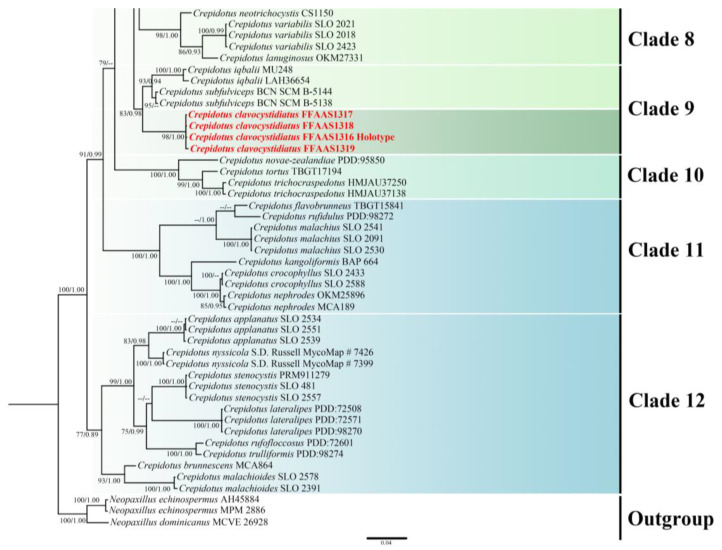
Phylogenetic tree inferred from the Bayesian Inference (BI) analysis based on a concatenated ITS and LSU dataset; bootstrap (BS) values over 75% and Bayesian posterior probabilities (BPP) over 0.90 are indicated. The new species are marked in red. In the top left corner, the figure caption indicates that different sections and series are marked with different colors.

The dataset matrix was composed of 68 taxa, 126 samples, 174 sequences (104 ITS, and 70 LSU). Among these, 144 sequences were downloaded from GenBank (89 ITS, and 55 LSU), and 30 sequences (15 ITS, and 15 LSU) were amplified in this study. *Neopaxillus dominicanus* Angelini & Vizzini and *N. echinospermus* (Speg.) Singer were selected as the outgroups [53]. The aligned length, including gaps, encompassed 1782 nucleotide sites, with 861 for ITS and 921 for LSU. For BI analysis, the best-fit substitution model for both ITS and LSU was GTR + I + G. BI analysis resulted in an average standard deviation of split frequencies = 0.005577, the effective sample size (ESS) was 1333.8, and the average Potential Scale Reduction Factor (PSRF) parameter values = 1.000 after 5,000,000 MCMC generations. For ML analysis, the best-fit substitution model selected for ITS was TVM + I + G4 and that for LSU was GTR + I + G4, with a final log-likelihood value of −14,404.410766. The average bootstrap value was 87.17%, indicating generally high confidence in the tree’s branches. The BI and ML trees exhibited similar topologies, and the BI tree is shown in Figure 1. Those nodes supported by bootstrap of at least 75% and posterior probabilities exceeding 0.90 were incorporated.

According to the phylogenetic tree in Figure 1, twelve high-support clades were delineated. Clade 1 comprises eight species: *C. striatus* T. Bau & Y.P. Ge, *C. heterocystidiosus* T. Bau & Y.P. Ge, *C. dentatus* T. Bau & Y.P. Ge, *C. alabamensis* Murrill, *C. pseudomollis* T. Bau & Y.P. Ge, *C. fraxinicola* Murrill, *C. mollis* (Schaeff.) Staude, and *C. calolepis* (Fr.) P. Karst. This clade has strong support in BI analysis (BS/BPP = --/1.00); the species in this clade exhibit smooth elongated spores and their tissues do not have clamp connections. Based on Consiglio and Setti’s classification, all eight species in this clade belong to *C.* subg. *Crepidotus* sect. *Crepidotus* [6]. Clade 2 includes *C. wasseri* Kapitonov, Biketova, Zmitr. & Á. Kovács and *C. lamellomaculatus*, with strong support (BS/BPP = 97/1.00). The new species *C. lamellomaculatus* forms a well-supported clade (BS/BPP = 98/1.00).Both *C. wasseri* and *C. lamellomaculatus* possess smooth elongated spores and clamp connections [52]. Typically, species in *Crepidotus* with clamp connections exhibit ornamented basidiospores [4,6,12]. However, some species exhibit smooth basidiospores and clamp connections simultaneously, such as *C. wasseri* and *C. lamellomaculatus* in Clade 2, and *C. novae-zealandiae* Pilát, *C. tortus* A.M. Kumar & C.K. Pradeep, and *C. trichocraspedotus* T. Bau & Y.P. Ge in Clade 10 [11,12,34,52]. According to Consiglio & Setti’s classification, Clade 2 and Clade 10, characterized by clamp connections and smooth elongated spores, should be classified within *C.* subg. *Dochmiopus* sect. *Autochthoni* (Sennn-Irlet) Consiglio & Setii [6]. However, the current phylogenetic tree does not support this classification. Additionally, Clade 2 and Clade 10 exhibit significant differences in the morphology of their cheilocystidia. In Clade 2, the cheilocystidia of *C. wasseri* and *C. lamellomaculatus* are both lageniform and ventricose, whereas in Clade 10, the cheilocystidia are sinuously cylindrical to vine-like [52]. Clade 3 comprises *C. caspari* Velen., *C. inhonestus* P. Karst., *C. sphaerosporus* (Pat.) J.E. Lange, *C. circinatus* Hesler & A.H. Sm., *C. ulmicola* T. Bau & Y.P. Ge, *C. occidentalis* Hesler & A.H. Sm., and *C. fragilis* Joss. Among these, *C. occidentalis* and *C. fragilis* are classified in *C.* subg. *Dochmiopus* sect. *Autochthoni*, due to their smooth basidiospores and clamp connections [5,6]. The remaining taxa belong to *C.* subg. *Dochmiopus* sect. *Dochmiopus*. Specifically, *C. sphaerosporus* and *C. circinatus* are part of *C.* subg. *Dochmiopus* sect. *Dochmiopus* ser. *Dochmiopus* Consiglio & Setii, whereas *C. caspari*, *C. inhonestus* and *C. ulmicola* are classified in *C.* subg. *Dochmiopus* sect. *Dochmiopus* ser. *Caspari* Consiglio & Setii [4,6,20]. Although *C. ulmicola* was initially placed in *C.* subg. *Dochmiopus* sect. *Dochmiopus* ser. *Dochmiopus* when published, its smooth to nearly smooth basidiospores suggest a reclassification to *C.* subg. *Dochmiopus* sect. *Dochmiopus* ser. *Caspari* [20]. Additionally, *C. sphaerosporus* has been accepted as C. *cesatii* (Rabenh.) Sacc., and *C. fragilis* was combined into *C. autochthonus* [1,6]. Clade 4 includes three species, *C. subverrucisporus* Pilát, *C. herbaceus* T. Bau & Y.P. Ge and *C. palodensis* C.K. Pradeep & A.M. Kumar, all of which are regarded as *C.* subg. *Dochmiopus* sect. *Dochmiopus* ser. *Caspari*. These species are characterized by clavate cheilocystidia and a gregarious habit [1,10,20]. Clade 5 incorporates the new species *C. succineus* (BS/BPP = 100/1.00) along with nine other taxa. Except for *C. roseus* Singer and *C. epibryus* (Fr.) Quél., other taxa in this clade belong to *C.* subg. *Dochmiopus* sect. *Dochmiopus* ser. *Dochmiopus*. *Crepidotus roseus* is classified in *C.* subg. *Dochmiopus* sect. *Sphaeruli* due to its nearly globose basidiospores (Q_m_ < 1.10) [6,11]. As *C. epibryus* has smooth elongated spores and absent clamp connections, it is placed in *C.* subg. *Crepidotus* sect. *Versuti* Hesler & A.H. Sm.; *C. versutus* (Peck) Sacc. also belongs to this section for similar reasons [5,6]. Clade 5 contains several brightly colored species, including the yellow-to-orange *C. tobolensis* Kapitonov, Biketova & Zmitr., *C. succineus*, *C. praecipuus* E. Horak, *C. lutescens* T. Bau & Y.P. Ge, *C. yuanchui* Q. Na, Z.W. Liu & Y.P. Ge, and pink-to-red-colored *C. innuopurpureus* McMull.-Fish., T. Lebel & Senn-Irlet, and *C. roseus*. In Clade 6, except *C. cinnabarinus* Peck, the other taxa belong to *C.* subg. *Dochmiopus* sect. *Dochmiopus* ser. *Dochmiopus*, and *C. kauffmanii* Hesler & A.H. Sm., *C. reticulatus* T. Bau & Y.P. Ge and *C. cinnabarinus* are notably colored species. *Crepidotus cinnabarinus* Peck is classified in *C.* subg. *Crepidotus* sect. *Cinnabarinus* Hesler & A.H. Sm., due to its ornamented basidiospores and absence of clamp connections [6,27]. Additionally, we concur with the assessment by Jančovičová et al. regarding the GenBank accession numbers for *C. reticulatus* [60]. Upon reviewing the original publication, we confirmed that the holotype of *C. reticulatus* is correctly associated with the GenBank sequence MF461346. The sequence was previously misassigned, and we have submitted a correction request to GenBank to rectify this error. Among Clade 5 and Clade 6, *C. epibryus*, *C. versutus* and *C. cinnabarinus*, which present absent clamp connections, cannot be classified in *C.* subg. *Crepidotus* based on the current phylogenetic analysis. This suggests that the absence of clamp connections is not an exclusive characteristic of *C.* subg. *Crepidotus*, and other subgenera also include taxa without clamp connections. Therefore, the defining characteristic of *C.* subg. *Crepidotus* requires further study and refinement. All species in Clade 7 belong to *C.* subg. *Dochmiopus* sect. *Dochmiopus* ser. *Dochmiopus*. Within this clade, the new species *C. capitatocystidiatus* is well supported (BS/BPP = 100/1.00). *Crepidotus capitatocystidiatus* clusters with *C. affinis* E. Horak and *C. volubilis* C.K. Pradeep & A.M. Kumar, sharing similar guttiform basidiospores covered by irregularly punctate-verrucae [10,34]. Notably, *C. asiaticus* Guzm.-Dáv., C.K. Pradeep & T.J. Baroni in Clade 7, *C. iqbalii* A. Izhar, Usman & Khalid and *C. subfulviceps* (Murrill) Aime, Vila & P.-A. Moreau in Clade 9 differ from the typical *Crepidotus* species due to their agaricoid basidiomata, representing the stipitate group within *Crepidotus.* Clade 8 comprises three species within *C.* subg. *Dochmiopus* sect. *Dochmiopus* ser. *Dochmiopus*, characterized by their white basidiomata with a finely villous pileus surface. Clade 9 includes three species, *C. iqbalii*, *C. subfulviceps* and *C. clavocystidiatus.* The new species *C. clavocystidiatus* is strongly supported (BS/BPP = 98/1.00). *Crepidotus clavocystidiatus* belongs to *C.* subg. *Dochmiopus* sect. *Dochmiopus* ser. *Caspari*, and the other two species are classified in *C.* subg. *Dochmiopus* sect. *Dochmiopus* ser. *Dochmiopus*. The taxa of Clade 11 and Clade 12 all possess clamp connections and globose ornamented basidiospores and are classified as *C.* subg. *Dochmiopus*. sect. *Sphaeruli*. But in the phylogenetic tree, Clade 11 and Clade 12 do not form a monophyletic group, indicating that *C.* subg. *Dochmiopus* sect. *Sphaeruli* is polyphyletic. This aligns with the viewpoint proposed by Ge, which is further corroborated by our current phylogenetic analysis [61].

### 3.2. Taxonomy

**Figure 2:**
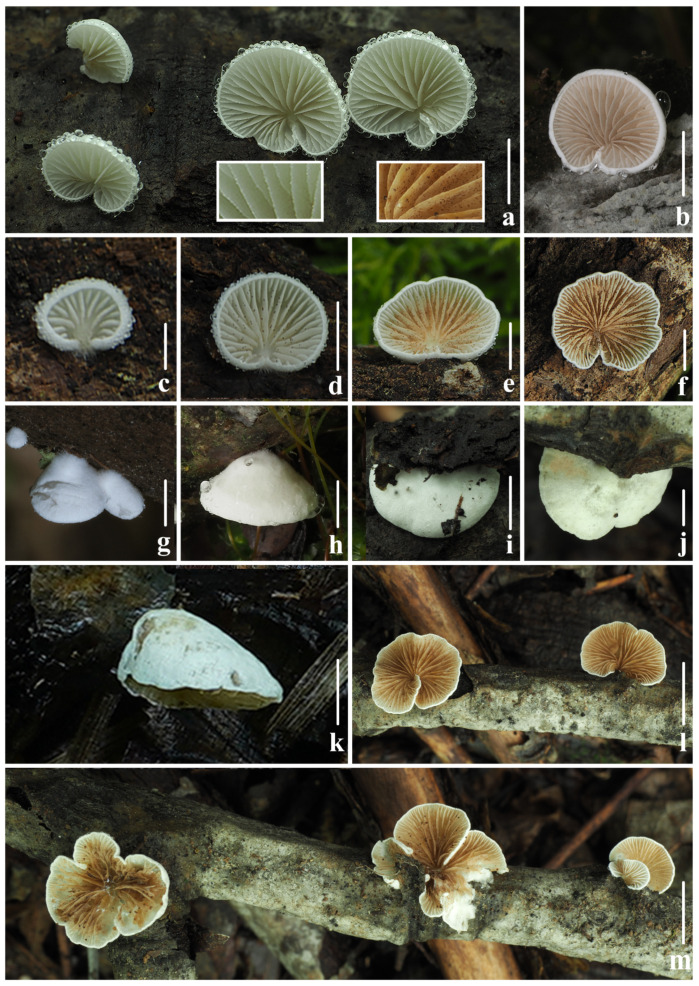
Fresh basidiomata of *Crepidotus lamellomaculatus* sp. nov. (**a**,**i**) *FFAAS1307*; (**b**,**g**) *FFAAS1305* (holotype); (**c**–**f**) *FFAAS1306*; (**h**,**k**) *FFAAS1309*; (**j**,**l**,**m**) *FFAAS1308*; (**a**) lamellae edge when young and matured. Bars: (**a**,**b**,**d**–**f**,**h**–**k**) = 5 mm; (**c**,**g**) = 2 mm; (**l**,**m**) = 10 mm. Photos by Yupeng Ge and Menghui Han.

**Figure 3:**
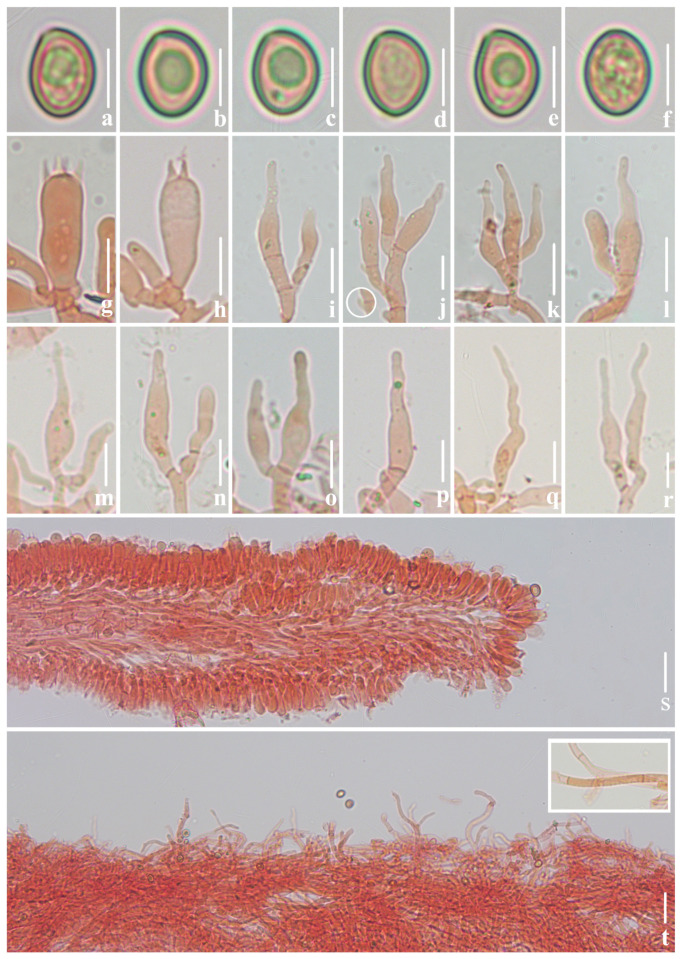
Microscopic features of *Crepidotus lamellomaculatus* (*FFAAS1305,* holotype). (**a**–**e**) Lateral view of basidiospores; (**f**) frontal view of basidiospores; (**g**,**h**) basidia; (**i**–**r**) cheilocystidia; (**j**) clamp connection; (**s**) lamellae trama; (**t**) pileipellis, encrusted hyphae. Bars: (**a**–**f**) = 5 μm; (**g**–**r**) = 10 μm; (**s**,**t**) = 30 μm. Structures (**a**–**f**) were rehydrated in 5% KOH aqueous solution and (**g**–**t**) were stained in 1% Congo red aqueous solution.

**Figure 4:**
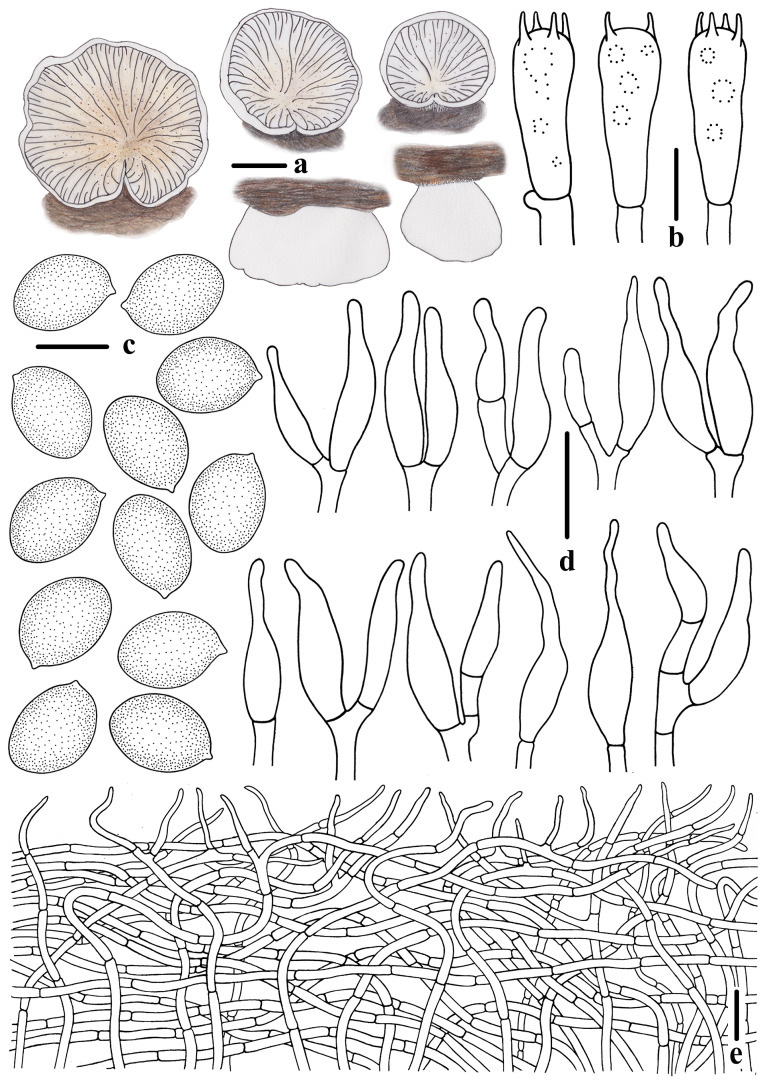
Morphological features of *Crepidotus lamellomaculatus* (*FFAAS1305,* holotype). (**a**) Basidiomata; (**b**) basidia; (**c**) basidiospores; (**d**) cheilocystidia; (**e**) pileipellis. Bars: (**a**) = 3 mm; (**b**) = 10 μm; (**c**) = 5 μm; (**d**,**e**) = 20 μm. Drawing by Menghui Han.

***Crepidotus lamellomaculatus*** M.H. Han, Q. Na, Y.P. Hu & Y.P. Ge, sp. nov., Figure 2, Figure 3 and Figure 4.

MycoBank no: 854914

Etymology: The epithet *lamellomaculatae* combines the Latin word ‘*lamellae*’ and the Latin adjective ‘*maculatae*’, meaning spotted. This name accurately describes the lamellae surface, which shows a few dark spots when matured.

Holotype: China. Hubei Province, Yichang City, Wufeng County, 19 June 2024, leg. Menghui Han, Jingwen Guo, Guanyu Qiu, Lijun Wang, Qin Na, and Yupeng Ge, 553 m asl, *FFAAS1305* (collection number NJ5410).

Diagnosis: Pileus white, covered by short white felted tomentum, margin incurved, lamellae edge nearly smooth when matured, stipe observed in early stage; basidiospores (5.6)6.0–6.5–7.1(7.6) × (4.2)4.4–4.8–5.2(5.7) μm, broadly ellipsoid to ellipsoid, smooth, cheilocystidia narrowly lageniform with a curved elongated neck, ventricose. Differs from *C. wasseri* by smaller cheilocystidia and basidiospores, and absent of pileocystidia.

Description: Pileus 4–18 mm broad, in young stage conchoidal to flabelliform, White (LIII), when matured semicircular to reniform, sometimes petaloid, nearly applanate, White (LIII), when old or dry Pale Olive Buff (XL21‴*f*), margin incurved in all stages; dry, covered by short White (LIII) felted tomentum, margin glabrescent, non-striated, not hygrophanous, attached laterally or dorsally to the substratum, White (LIII) villosity observed near the point of attachment. Lamellae less than 1mm broad, *L* = 12–17, *l* = 3–7, subdecurrent, ventricose, when young White (LIII), edge slightly fimbriated, when matured Isabella Color (XXX19″*i*) to Light Brownish Olive (XXX19″*k*), surface with a few dark spots, and edge nearly smooth. Stipe observed in young stage, clavate to cylindrical, lateral, White (LIII), with age persisting as a knob in lateral, indistinct. Context thin (<5 mm thick), White (LIII). Odor and taste not distinctive.

Basidiospores (417/8/5)(5.6)6.0–6.5–7.1(7.6) × (4.2)4.4–4.8–5.2(5.7) μm, Q = (1.24)1.26–1.46(1.50), Q_m_ = 1.35 ± 0.061, [HOLOTYPE (68/2/1)(6.2)6.3–6.8–7.2(7.4) × (4.3)4.6–4.9–5.2(5.3) μm, Q = (1.30)1.32–1.47(1.49), Q_m_ = 1.39 ± 0.039], broadly ellipsoid to ovoid in frontal view, broadly ellipsoid to ellipsoid in lateral view, greyish orange to yellowish brown in 5% KOH aqueous solution, smooth (under oil), sometimes granular contents or large oil drop observed. Basidia 16–25 × 5–7 μm, short clavate, apex obtuse, base slightly constricted, four-spored, rarely two-spored, sterigmata 1.9–3.8 μm long, thin-walled (<0.5 μm thick), hyaline. Pleurocystidia absent. Cheilocystidia 17–41 × 3–6 μm, slightly clavate to utriform, narrowly lageniform with a curved neck, apex rounded, the middle part ventricose and slightly attenuated at the base, clumped, hyaline, thin-walled (<0.5 μm thick). Pileipellis a tomentum, composed of interwoven long cylindrical hyphae, 3–6 μm in diameter, sometimes branched, terminal cells flexuous, sometimes vertical to the pileus, thin-walled (<0.5 μm thick), hyaline, non-gelatinized. Lamellae trama subregular, composed of cylindrical hyphae, 3–6 μm in diameter, interwoven, at times inflated, non-gelatinized. Clamp connections present only in basidia and hymenium, occasionally observed in cheilocystidia.

Habit and habitat. Scattered on rotten branches and woods in the mixed broadleaf-conifer forests of temperate and subtropical zones, mainly found under *Betula* sp., *Castanopsis* sp., *Cypressi* sp., *Cunninghamia* sp., and *Fraxinus* sp.

Other specimens examined: China. Jilin Province, Yanbian Korean Autonomous Prefecture, Antu County, Changbaishan National Nature Reserve, 16 August 2021, leg. Menghui Han, Zewei Liu, Qin Na, and Yupeng Ge, 831m asl, *FFAAS1306* (collection number GN1378), same location, 17 August 2021, leg. Menghui Han, Zewei Liu, Qin Na, and Yupeng Ge 843 m asl, *FFAAS1307* (collection number GN1398), 841 m asl, *FFAAS1308* (collection number GN1399); Heilongjiang Province, Hegang City, Luobei County, Taipinggou National Natural Reserve, 6 July 2023, leg. Menghui Han, Renxiu Wei, Tingting Sun, Zengcai Liu, Weiguo Chen, Xinyu Tong, Yawei Li, Nannan Geng, Li Zou, Qin Na, and Yupeng Ge, 476 m asl, *FFAAS1309* (collection number GN1878).

Notes: Within the genus *Crepidotus*, the species with clamp connections typically exhibit ornamented basidiospores, while those lacking clamp connections generally present smooth basidiospores [1,5,6]. However, *C. lamellomaculatus* is an exceptional species within the genus, possessing both clamp connections and smooth basidiospores, and it is considered to belong to *C*. subg. *Dochmiopus* sect. *Autochthoni.* Before that, the only species in this section was *C. autochthonus*; it was distinguished from *C. lamellomaculatus* by bigger basidiospores (7.0–9.0 × 5.0–6.0 μm or 7.1–8.5 × 4.9–5.7 μm) and cylindrical to narrowly utriform cheilocystidia [1,6]. Phylogenetic analysis showed the *C. wasseri* is most related to *C. lamellomaculatus*, and there are many similarities in macroscopical, but *C. wasseri* can be distinguishable by its narrowly lageniform pileocystidia, bigger cheilocystidia [(33.1)37.1–59.6(74.0) × (3.9)4.6–7.3(8.4) μm] and basidiospores [(6.5)7.1–8.6(9.0) × (4.5)4.8–5.5(5.9) μm, av. = 7.8 × 5.2 μm] [50]. *Crepidotus trichocraspedotus* is a species discovered in China, possessing smooth basidiospores and clamp connections similar to *C. lamellomaculatus*, but it can be easily distinguished from bigger basidiospores [(8.8)9.1–10.5(10.7) × 6.0–6.8(7.1) μm], vine-shape with bifurcation cheilocystidia and a trichoderm pileipellis [12]. *Crepidotus tortus* and *C. novae-zelandiae* Pilát are characterized by white woolly-to-appressed squamulose pileus, smooth basidiospores, and clamp connections that resemble *C. lamellomaculatus*. However, both *C. tortus* and *C. novae-zelandiae* are distinguished from *C. lamellomaculatus* by possessing bigger basidiospores [*C. novae-zealandiae* (9.5)10–12 × (7)7.5–8 μm, *C. tortus* 8–10.5 × 5.5–6.5(6.8) μm] and flexuous cylindrical cheilocystidia [11,34].

**Figure 5:**
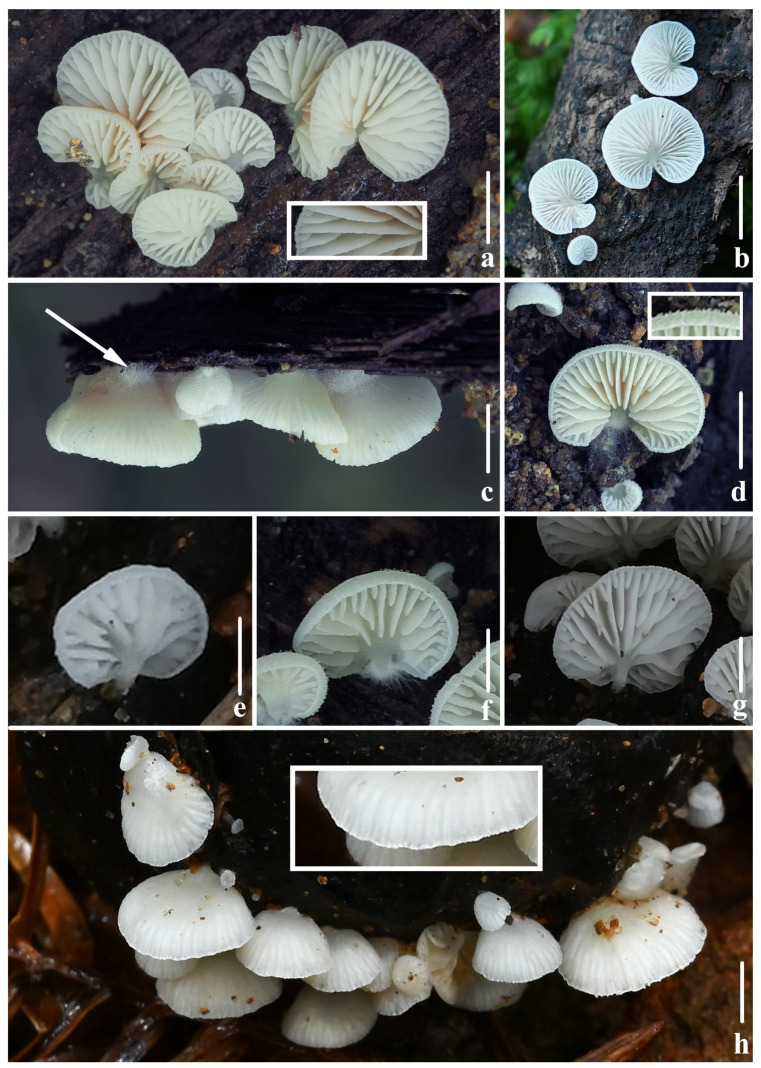
Fresh basidiomata of *Crepidotus capitatocystidiatus* sp. nov. (**a**,**c**,**d**,**f**) *FFAAS1311*; (**b**) *FFAAS1312*; (**e**,**g**,**h**) *FFAAS1310* (holotype); (**a**) lamellae edge fimbriated when matured; (**c**) clear villosity near the point of attachment; (**d**,**h**) pileus margin tomentose. Bars: (**a**–**d**,**h**) = 5 mm; (**e**–**g**) = 1 mm. Photos by Yupeng Ge, Junqing Yan and Menghui Han.

**Figure 6:**
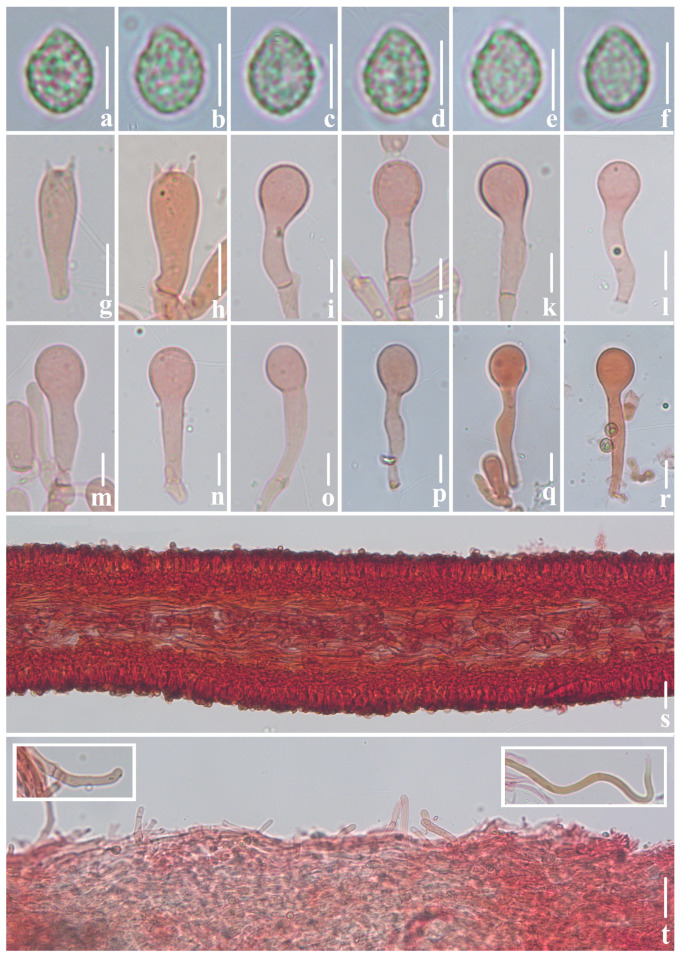
Microscopic features of *Crepidotus capitatocystidiatus* (*FFAAS1310*, holotype). (**a**–**e**) Lateral view of basidiospores; (**f**) frontal view of basidiospores; (**g**,**h**) basidia; (**i**–**r**) cheilocystidia; (**s**) lamellae trama; (**t**) pileipellis, clamp connection of pileipellis cell and oleiferous hyphae. Bars: (**a**–**f**) = 5 μm; (**g**–**r**) = 10 μm; (**s**,**t**) = 20 μm. Structures (**a**–**f**) were rehydrated in 5% KOH aqueous solution and (**g**–**t**) were stained in 1% Congo red aqueous solution.

**Figure 7:**
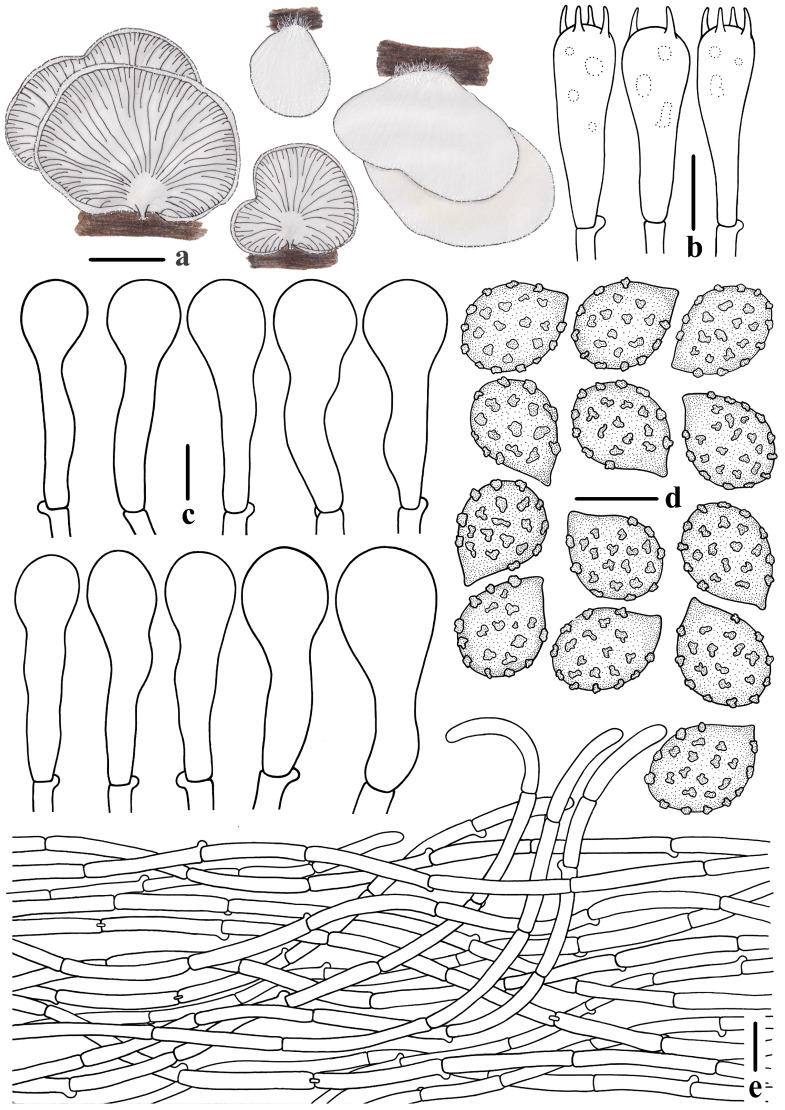
Morphological features of *Crepidotus capitatocystidiatus* (*FFAAS*1310, holotype). (**a**) Basidiomata; (**b**) basidia; (**c**) cheilocystidia; (**d**) basidiospores; (**e**) pileipellis. Bars: (**a**) = 5 mm; (**b**–**c**) = 10 μm; (**d**) = 5 μm; (**e**) = 20 μm. Drawing by Menghui Han.

***Crepidotus capitatocystidiatus*** M.H. Han, H. Zeng, Q. Na & Y.P. Ge, sp. nov., Figure 5, Figure 6 and Figure 7.

MycoBank no: 854915

Etymology: The epithet *capitatocystidiatus* derives from the Latin words ‘*capitata*’, meaning capitate, and ‘*cystidia*’. The name is chosen to describe the cheilocystidia apex globose to capitate.

Holotype: China. Fujian Province, Nanping City, Wuyishan National Park, 13 August 2021, leg. Menghui Han, Zewei Liu, Junqing Yan, Hui Zeng, Qin Na, and Yupeng Ge, 728 m asl, *FFAAS1310* (collection number NJ3779).

Diagnosis: Pileus white, margin tomentose, translucent striate, not hygrophanous; lamellae edge smooth, stipe present in young stage; basidiospores 4–6.2–7.3(7.7) × (4.1)4.3–5.0–5.7(6.2) μm, guttiform, covered by irregularly punctate-verrucae, cheilocystidia clavate with a globose apex, clamp connections present. Differs from *C. palmarum* Singer by pileus hygrophanous and distinguishable habitat.

Description: Pileus 6–25 mm broad, in young stage spatuliform, ungulate to conchoid, margin incurved, when matured flabelliform to rounded flabelliform, occasionally lobed, plano-convex, margin gradually straight; White (LIII), at times Chamois (XXX19″*b*) to Isabella color (XXX19″*i*) by basidiospores depositing on surface; White (LIII) pubescence when young, with age gradually sparse, when matured almost smooth, in all stages margin weakly tomentose; dry, translucent striate, not hygrophanous; pileus attached almost laterally, but some dorsally to the substratum, clear villosity near the point of attachment. Lamellae 0.5–1 mm broad, *L* = 11–23, *l* = 3–7, subdecurrent to decurrent, subventricose, edge weakly fimbriated; White (LIII) when young, becoming *Buff-Yellow (IV19*d*) to Baryta Yellow (IV21*f*), when matured Orange-Citrine (IV19*k*) to Buckthom Brown (XV17′*i*). Stipe present when young, cylindrical, when matured as a lateral knob or covered by lamellae, subtransparent. Context thin (<5 mm thick), White (LIII), hyaline. Odor and taste not distinctive.

Basidiospores (141/4/3)(5.3)5.4–6.2–7.3(7.7) × (4.1)4.3–5.0–5.7(6.2) μm, Q = (1.13)1.20–1.35(1.40), Q_m_ = 1.26 ± 0.051, [HOLOTYPE (80/2/1)(5.3)5.4–5.9–6.5(7.4) × (4.2)4.3–4.7–5.4(5.8) μm, Q = (1.13)1.19–1.35(1.40), Q_m_ = 1.25 ± 0.051], broadly fusiform to ovoid in frontal view, broadly ellipsoid to guttiform bottom slightly pointed in lateral view, greyish yellow to olive brown in 5% KOH aqueous solution, irregularly punctate-verrucose to verrucose (ornamentation verrucose type I), suprahilar depression weakly developed (under oil). Basidia 19–26 × 6–8 μm, clavate to broadly clavate, slightly constricted towards the base, four-spored, rarely two-spored, sterigmata 1.8–4.5 μm long, thin-walled (<0.5 μm thick), hyaline. Pleurocystidia absent. Cheilocystidia 29–60 × 8–18 μm, clavate to narrowly clavate with a globose to ovoid apex, middle portion more or less ventricose, constrict to base, clumped, hyaline, thin- to thick-walled (≤0.6 μm thick). Pileipellis a rectocutis, composed of cylindrical parallel hyphae, 4–8 μm diameter, sometimes interwoven, occasionally branched, thin-walled (<0.5 μm thick); some terminal cells semierect and bundled, contributed to the formation of short pubescence on pileus surface, hyaline, not-gelatinized, oleiferous hyphae observed occasionally. Lamellae trama subregular, composed of subcylindrical hyphae, 6–11 μm diameter, nearly parallel arrangement, more or less inflated, non-gelatinized. Clamp connections present in all tissues.

Habit and habitat: gregarious on deciduous twigs, rotten woods, and nutshell in mixed broadleaf-conifer forests of temperate and subtropical zones, mainly found under *Betula* sp., *Tilia* sp., *Larix* sp., and *Pinus* sp.

Other specimens examined: China. Fujian Province, Quanzhou City, Dehua County, Lingjiao Village, 30 April 2021, leg. Menghui Han, Zewei Liu, Hui Zeng, Qin Na, and Yupeng Ge, 457 m asl, *FFAAS1311* (collection number GN0812); Jilin Province, Tonghua City, Baijifeng National Forest Park, 26 June 2023, leg. Menghui Han, Xiaoliang Liu, Renxiu Wei, Qin Na, and Yupeng Ge, 588 m asl, *FFAAS1312* (collection number GN1547).

Notes: *Crepidotus capitatocystidiatus* is classified within *C.* subg. *Dochmiopus* sect. *Dochmiopus* ser. *Dochmiopus* due to its verrucose basidiospores and clamped hyphae [6]. Within this series, *C. cesatii* bears a resemblance to *C. capitatocystidiatus*, but it can be distinguished by its pileus margin striated, fimbriated lamellae edge, and irregular cheilocystidia, which generally knobby or branched [1,6]. *Crepidotus palmarum* also shares many similarities with *C. capitatocystidiatus* due to guttiform basidiospores and capitate cheilocystidia. However, *C. palmarum* is macroscopically distinct by its hygrophanous pileus, smooth margin, and sessile. Furthermore, *C. palmarum* and *C. capitatocystidiatus* exhibit significant differences in habitat and distribution. *Crepidotus palmarum* was recorded in tropical and subtropical America, gregarious on leaves and leaf petioles of *Trachycarpus* sp. or rotten trunk of *Quercus* sp., whereas *C. capitatocystidiatus* is a species that occurred in temperate and subtropical China, gregarious on rotten branches and woods of *Betula* sp., *Tilia* sp., *Larix* sp., and *Pinus* sp. [27,62]. Additionally, *C. affinis* and *C. volubilis* are close to the new species in phylogeny analysis, while *C. affinis* can be distinguished by its globose basidiospores and slender cheilocystidia [(35)45–65 × 5–10 μm] [34]. *Crepidotus volubilis* is different from the new species in ventricose cheilocystidia without capitate and pileus margin straight to slightly upturned [10].

**Figure 8:**
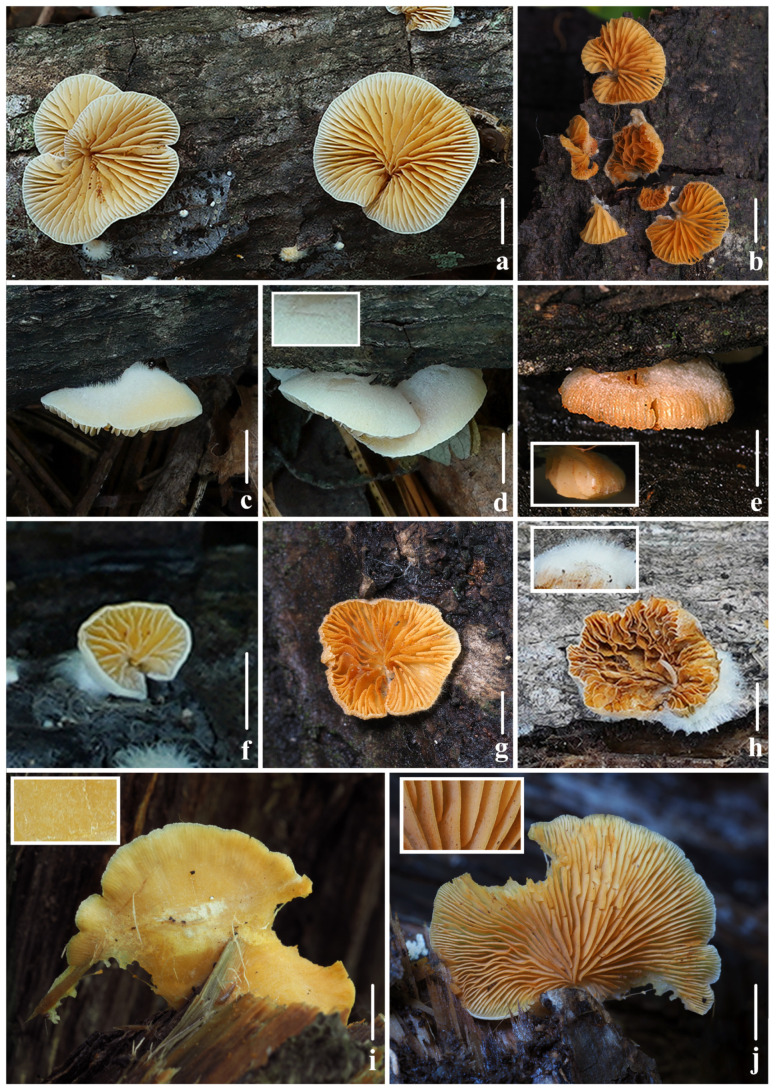
Fresh basidiomata of *Crepidotus succineus* sp. nov. (**a**,**c**,**d**,**f**,**h**) *FFAAS1313* (holotype); (**b**,**e**,**g**) *FFAAS1315*; (**i**,**j**) *FFAAS1314*; (**d**) short tomentums when immature in pileus; (**e**) lamellae in side view; (**h**) tomentums near the point of attachment; (**i**) pubescence when matured on pileus; (**j**) lamellae edge smooth when matured. Bars: (**a**,**c**–**f**,**h**–**j**) = 5 mm; (**b**,**g**) = 10 mm. Photos by Yupeng Ge and Menghui Han.

**Figure 9:**
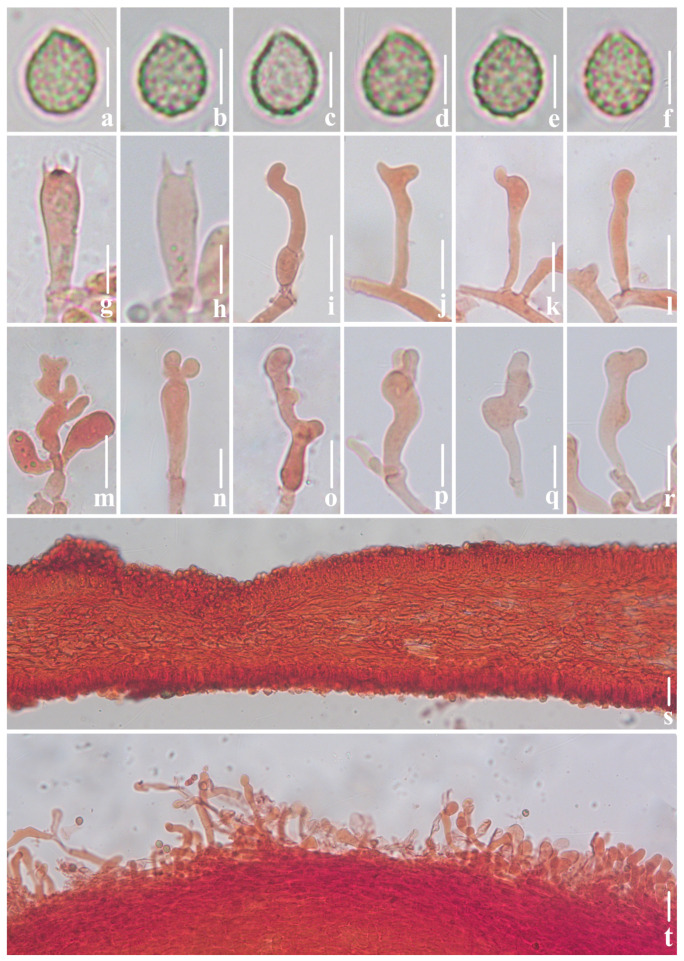
Microscopic features of *Crepidotus succineus* (*FFAAS*1313, holotype). (**a**–**e**) Lateral view of basidiospores; (**f**) frontal view of basidiospores; (**g**,**h**) basidia; (**i**–**l**) pileocystidia; (**m**–**r**) cheilocystidia; (**s**) lamellae trama; (**t**) pileipellis. Bars: (**a**–**f**) = 5 μm; (**g**,**h**) = 10 μm; (**i**–**t**) = 20 μm. Structures (**a**–**f**) were rehydrated in 5% KOH aqueous solution and (**g**–**t**) were stained in 1% Congo red aqueous solution.

**Figure 10:**
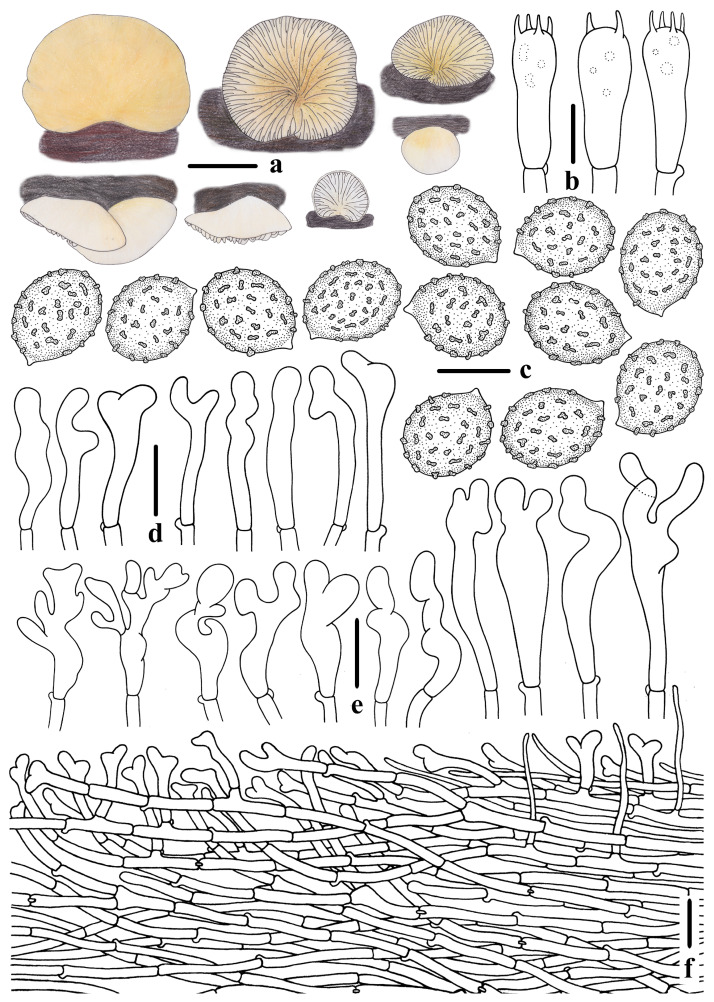
Morphological features of *Crepidotus succineus* (*FFAAS*1313, holotype). (**a**) Basidiomata; (**b**) basidia; (**c**) basidiospores; (**d**) pileocystidia; (**e**) cheilocystidia; (**f**) pileipellis. Bars: (**a**) = 10 mm; (**b**) = 10 μm; (**c**) = 5 μm; (**d**–**f**) = 20 μm. Drawing by Menghui Han.

***Crepidotus succineus*** M.H. Han, L. Zou, Q. Na & Y.P. Ge, sp. nov., Figure 8, Figure 9 and Figure 10.

MycoBank no: 854916

Etymology: The epithet is from the Latin word ‘*succineus*’, meaning amber. The name reflects the color of the basidiomata.

Holotype: China. Heilongjiang Province, Hegang City, Luobei County, Taipinggou National Natural Reserve, 5 July 2023, leg. Menghui Han, Renxiu Wei, Tingting Sun, Zengcai Liu, Weiguo Chen, Xinyu Tong, Yawei Li, Nannan Geng, Li Zou, Qin Na, and Yupeng Ge, 454 m asl, *FFAAS1313* (collection number GN1824).

Diagnosis: Pileus buff yellow to apricot yellow, covered by short villosity, margin straight, translucent striate, not hygrophanous; basidiospores (5.2)5.7–6.3–7.0(7.4) × (4.5)4.7–5.3–5.8(6.0) μm, guttiform and verrucose, cheilocystidia usually irregularly knobbed in upper portion, pileipellis a clavicutis, flexuous clavate pileocystidia observed. Differs from *C. praecipuus* by variable cheilocystidia and a clavicutis pileipellis with pileocystidia.

Description: Pileus 6–26 mm broad, in young stage spathuliform to rounded reniform to petaloid, margin weakly incurved, Naphthalene Yellow (XVI23′*f)* to *Cream Color (XVI19′*f)*, with age flabelliform to semicircular, when matured applanate and margin progressively straight, *Buff Yellow (IV19*d*) to Apricot Yellow (IV19*b*); dry, covered by White (LIII) short tomentum when immature, transitioning to slight pubescence when matured, translucent striate, not hygrophanous; mostly dorsally attached to the substratum, attached point covered by White (LIII) tomentum. Lamellae less than 2 mm broad, *L* = 9–20, *l* = 3–9, adnate to subdecurrent, ventricose, when young *Cream Color (XVI19′*f*) to *Naples Yellow (XVI19′*d*), edge slightly fimbriated, when matured *Buff Yellow (IV19*d*) to Mustard Yellow (XVI19′*b*), surface with a few dark spots, edge smooth. Stipe obvious when young, nearly cylindrical, White (LIII), subtransparent, when matured becoming an eccentric knob in lateral. Context thin (<5 mm thick), subtranslucent. Odor and taste not distinctive.

Basidiospores (238/5/3)(5.2)5.7–6.3–7.0(7.4) × (4.5)4.7–5.3–5.8(6.0) μm, Q =(1.09)1.13–1.27(1.38), Q_m_ = 1.20 ± 0.045, [HOLOTYPE (146/3/1)(5.2)5.7–6.3–6.9(7.4) × (4.5)4.8–5.3–5.8(6.0) μm, Q = (1.09)1.12–1.26(1.38), Q_m_ = 1.19 ± 0.043], subglobose to broadly ellipsoid in frontal view, broadly ellipsoid to guttiform in lateral view, greyish yellow to olive brown in 5% KOH aqueous solution, irregularly punctate-verrucose to verrucose (ornamentation verrucose type I), suprahilar depression weakly developed (under oil). Basidia 18–26 × 5–8 μm, clavate, gradually constricted to base, four-spored, rarely two-spored, sterigmata 2.2–4.2 μm long. Pleurocystidia absent. Cheilocystidia 27–70 × 3–7 μm, predominantly contorted narrowly utriform to slender clavate, bifurcated, digitiform to irregularly knobbed branching at middle part and apex, occasionally coralline in densely branched specimens, and taper towards a constricted base, hyaline, thin-walled (<0.5 μm thick). Pileipellis a clavicutis, composed of cylindrical hyphae, 4–7 μm diameter, sometimes interwoven, occasionally branched, nearly hyaline, thin-walled (<0.5 μm thick), not-gelatinized; a few terminal cells slender and semierect, contributing to short villosity on pileus surface, majority terminal cells form pileocystidia, 24–63 × 3–9 μm, clavate, flexuous, sometimes the upper portion branched. Lamellae trama subregular, composed of cylindrical hyphae, 5–11 μm diameter, tightly arranged, slightly inflated, non-gelatinized. Clamp connections present in all tissues.

Habit and habitat: scattered on rotten branches in temperate mixed broadleaf-conifer forests, mainly found under *Betula* sp., *Fraxinus* sp., *Pinus* sp., and *Tilia* sp.

Other specimens examined: China. Jilin Province, Yanbian Korean Autonomous Prefecture, Antu County, Changbaishan National Nature Reserve, 28 June 2023, leg. Menghui Han, Renxiu Wei, Bai Wang, Qin Na, and Yupeng Ge, 1309 m asl, *FFAAS1314* (collection number GN1064). Heilongjiang Province, Hegang City, Luobei County, Taipinggou National Natural Reserve, 6 July 2023, leg. Menghui Han, Renxiu Wei, Tingting Sun, Zengcai Liu, Weiguo Chen, Xinyu Tong, Yawei Li, Nannan Geng, Li Zou, Qin Na, and Yupeng Ge, 462 m asl, *FFAAS1315* (collection number GN1917).

Notes: Due to clamp connections and irregularly punctate-verrucose to verrucose basidiospores, *C. succineus* is considered a member of *C.* subg. *Dochmiopus* sect. *Dochmiopus* ser. *Dochmiopus* [6]. In this series, *C. luteolus* Sacc. shares similar basidiomata, but it can be distinguished by oblong-to-cylindrical basidiospores (Q = 1.63–1.99 or Q = 1.55–2.10), cylindrical to narrowly lageniform cheilocystidia, and absent pileocystidia [6,62]. Phylogenetically, *C. praecipuus* is closed to the new species, resembling white tomentum at the point of attachment and guttiform basidiospores [34]. However, cheilocystidia of *C. praecipuus* are broadly clavate or vesiculose, and pileipellis is a cutis without pileocystidia [34]. *Crepidotus tobolensis* possesses similar basidiomata color and basidiospores to *C. succineus*, but is distinguished by hygrophanous pileus, denser lamellae, and a cutis pileipellis [50]. There are two species with similar basidiomata color to *C. succineus* from China, *C. yuanchui* and *C. lutescens*, but they can be easily distinguished by pileipellis type and morphology of cheilocystidia [4,7].

**Figure 11:**
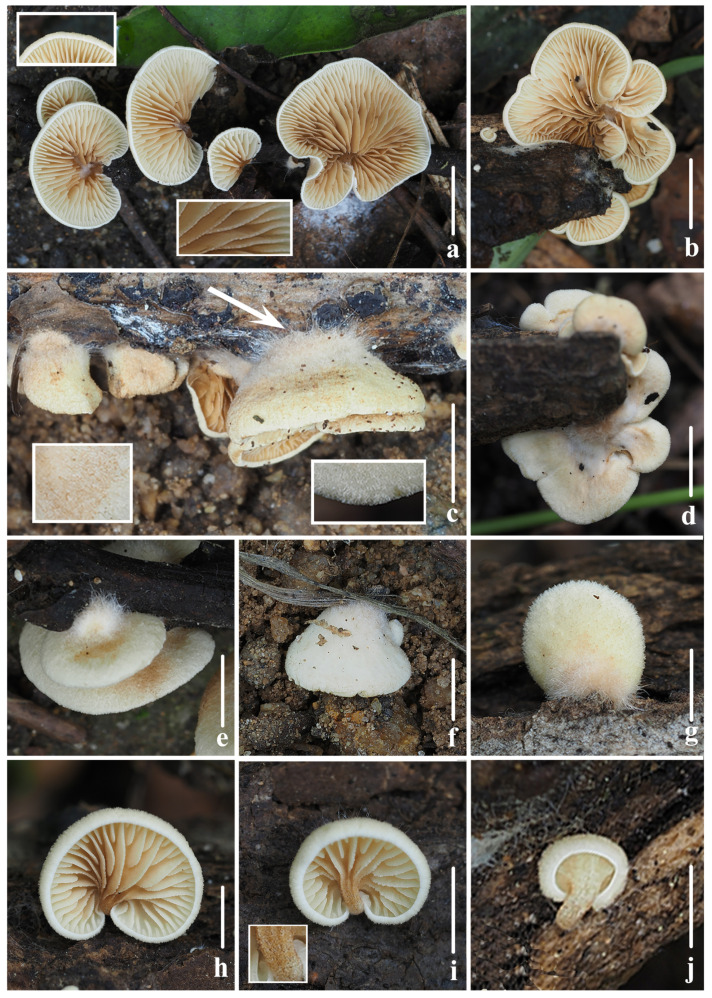
Fresh basidiomata of *Crepidotus clavocystidiatus* sp. nov. (**a**,**b**,**d**,**e**,**g**–**j**) *FFAAS1319*; (**c**,**f**) *FFAAS1316* (holotype); (**a**) lamellae edge fimbriated when matured; (**c**) villosity and tomentums on pileus surface; (**i**) stipe pruinose. Bars: (**a**–**d**) = 5 mm; (**e**–**i**) = 3 mm; (**j**) = 1 mm. Photos by Yupeng Ge and Menghui Han.

**Figure 12:**
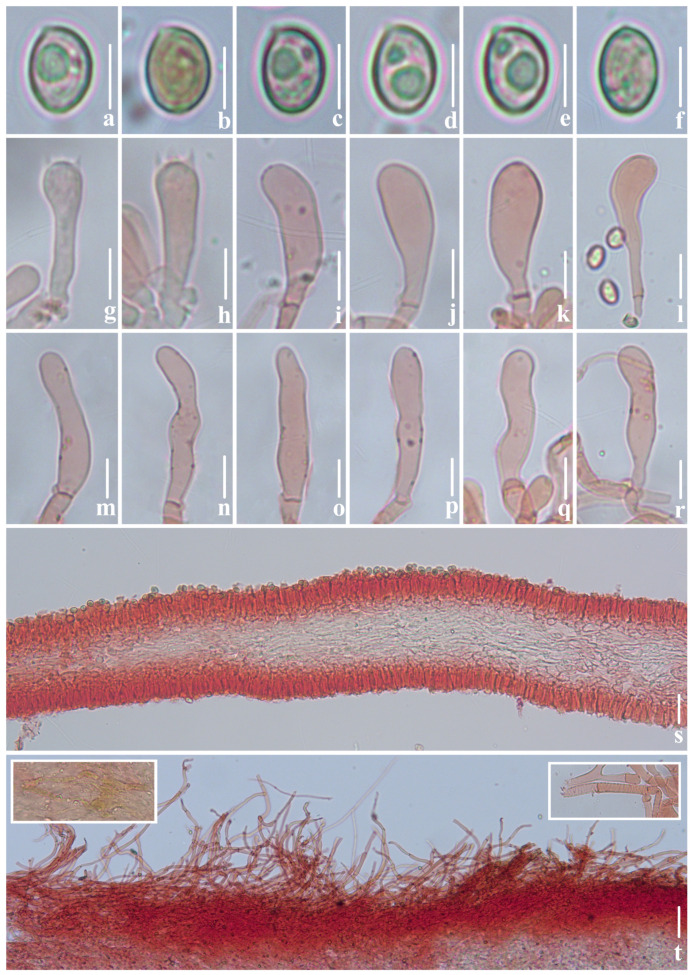
Microscopic features of *Crepidotus clavocystidiatus* (*FFAAS*1316, holotype). (**a**–**e**) Lateral view of basidiospores; (**f**) frontal view of basidiospores; (**g**,**h**) basidia; (**i**–**r**) cheilocystidia; (**s**) lamellae trama; (**t**) pileipellis, pigment and encrusted hyphae. Bars: (**a**–**f**) = 5 μm; (**g**–**r**) = 10 μm; (**s**,**t**) = 30 μm. Structures (**a**–**f**) were rehydrated in 5% KOH aqueous solution and (**g**–**t**) were stained in 1% Congo red aqueous solution.

**Figure 13:**
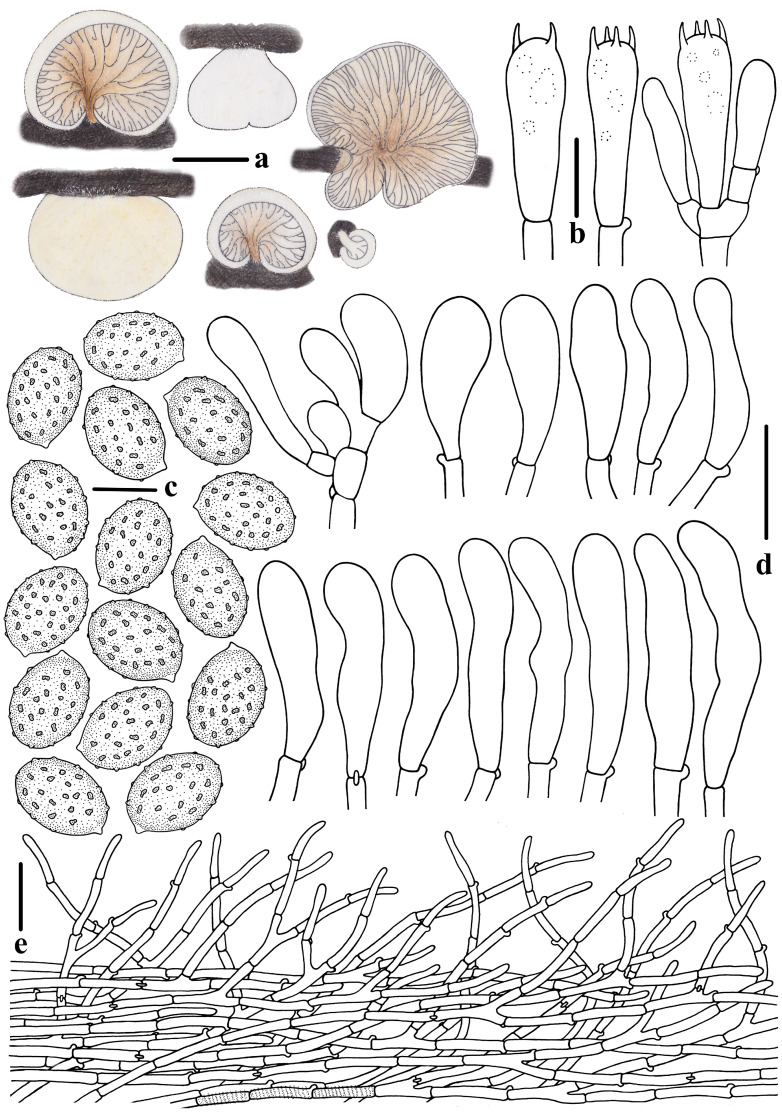
Morphological features of *Crepidotus clavocystidiatus* (*FFAAS*1316, holotype). (**a**) Basidiomata; (**b**) basidia; (**c**) basidiospores; (**d**) cheilocystidia; (**e**) pileipellis. Bars: (**a**) = 5 mm; (**b**) = 10 μm; (**c**) = 5 μm; (**d**,**e**) = 20 μm. Drawing by Menghui Han.

***Crepidotus clavocystidiatus*** M.H. Han, Q. Na & Y.P. Ge, sp. nov., Figure 11, Figure 12 and Figure 13.

MycoBank no: 854917

Etymology: The epithet *clavocystidiatus* combines the Latin adjective ‘*clavatus*’ and the Latin word ‘*cystidia*’. This name describes the shape of the cheilocystidia, which is clavate to narrowly clavate.

Holotype: China. Shandong Province, Tai’an City, Culaishan National Forest Park, 23 July 2021, leg. Menghui Han, Zewei Liu, Yulan Sun, Qin Na, and Yupeng Ge, 883 m asl, *FFAAS1316* (collection number GN1297).

Diagnosis: Pileus ivory yellow, covered by dense white flat villosity in all stages, white dense fibrillose hyphae observed on attachment of the substratum, non-striated; stipe present, pruinose; basidiospores (5.9)6.1–6.7–7.3(7.7) × (4.1)4.3–4.7–5.1(5.3) μm, smooth to faintly verrucose, pileipellis a rectocutis to plagiotrichoderm, clamp connections present. Differs from *C. caspari* var. *caspari* Velen. by colored stipe and smaller basidiospores.

Description: Pileus 5–14 mm broad, in young stage umbonate to conchoid or ungulate, White (LIII) to *Suphur Yellow (V25*f*), becoming flabelliform to semicircular, at times somewhat petaloid, when matured nearly applanate, Ivory Yellow (XXX21″*f*) to Naphthalene Yellow (XVI23′*f*), margin incurved, at times Isabella Color (XXX19″*i*) by basidiospores depositing on surface; dry, covered by White (LIII) flat villosity, margin weakly tomentose, non-striated, not hygrophanous, pileus attached laterally to the substratum, clearly fibrillose hyphae observed near the point of attachment. Lamellae less than 1 mm broad, *L* = 12–22, *l* = 3–9, free or subdecurrent, ventricose, edge fimbriated, when young Marguerite Yellow (XXX23″*f*) to Old Gold (XV19′*i*), becoming Pale Oliver-Buff (XL21″*f*) to Deep Olive-Buff (XL21″*b*), when matured Chamois (XXX19″*b*). Stipe observed in all stages, slightly clavate to cylindrical, lateral, *Olive-Buff (XL21″*d*) to Isabella Color (XXX19″*i*), pruinose, with age persisting as a bent cylinder or occasionally lateral knob. Context thin (<5 mm thick), White (LIII), subtranslucent. Odor and taste not distinctive.

Basidiospores (174/5/4)(5.9)6.1–6.7–7.3(7.7) × (4.1)4.3–4.7–5.1(5.3) μm, Q = (1.25)1.30–1.55(1.61), Q_m_ = 1.41 ± 0.074, [HOLOTYPE (77/2/1)(5.9)6.2–6.7–7.2(7.7) × (4.2)4.4–4.8–5.2(5.3) μm, Q = (1.25)1.31–1.52(1.59), Q_m_ = 1.40 ± 0.065], broadly ellipsoid to ellipsoid in frontal view, ovoid to ellipsoid in lateral view, greyish yellow to olive brown in 5% KOH aqueous solution, smooth to faintly verrucose (under oil, ornamentation rugulose-verruculose type I), sometimes granular contents or large oil drop are observed. Basidia 21–31 × 6–8 μm, clavate, four-spored, rarely two-spored, sterigmata 1.8–4.8 μm long, thin-walled (<0.5 μm thick), hyaline. Pleurocystidia absent. Cheilocystidia 28–45 × 6–12 μm, clavate to narrowly clavate, sometimes apex round or subcapitate, not forked, base contracted, flexuous, clumped, hyaline, thin-walled (<0.5 μm thick). Pileipellis a rectocutis to plagiotrichoderm, composed of densely arranged cylindrical hyphae, interwoven and branched, 5–9 μm wide, thin- to thick-walled (0.3–0.9 μm), weakly yellow-brown hyphae, some terminal cells erected, forming short-villosity on pileus surface, nearly hyaline, a few hyphae encrusted, non-gelatinized. Lamellae trama intermixed, composed of subparallel cylindrical hyphae 3–6 μm, more or less inflated, physalohyphae observed near trama base, 8–18 μm, measured up to 33 μm diameter, subregular, non-gelatinized. Clamp connections present in all tissues.

Habit and habitat: scattered on rotten twigs in temperate mixed broadleaf-conifer forests, mainly were found under *Fagaceae* sp., *Pinaceae* sp., *Populus* sp., and *Quercus* sp.

Other specimens examined: China. Shandong Province, Tai’an City, Culaishan National Forest Park, 23 July 2021, leg. Menghui Han, Zewei Liu, Yulan Sun, Qin Na, and Yupeng Ge, 909 m asl, *FFAAS1317*(collection number GN1294), 899 m asl, *FFAAS1318* (collection number GN1296); Shandong Province, Yantai City, Luoshan National Forest Park, 19 July 2021, Menghui Han, Zewei Liu, Yulan Sun, Qin Na, and Yupeng Ge, 223 m asl, *FFAAS1319* (collection number GN1157).

Notes: *Crepidotus clavocystidiatus* is classified into *C.* subg. *Dochmiopus* sect. *Dochmiopus* ser. *Caspari* by clamp connections and smooth to rugulose-verruculose basidiospores [6]. Within this series, *C. caspari* var. *caspari* and *C. subverrucisporus* share similar morphological characteristics to *C. clavocystidiatus*, but can be easily distinguished from *C. clavocystidiatus* by white to cream pileus and larger basidiospores (*C. caspari* var. *caspari* 7.1–8.9 × 4.6–5.6 μm, *C. subverrucisporus* 7.7–9.4 × 5.1–6.3 μm or (7.0)7.5–10.0(11.0) × (4.5)5.0–6.0(7.0) μm or 7.2–9.1 × 4.3–5.3 μm) [6,62,63]. In *C.* subg. *Dochmiopus* sect. *Dochmiopus* ser. *Caspari*, there is a species, *C. furcaticystidiosus* Q. Na, M.H. Han, R.X. Wei, H. Zeng & Y.P. Ge, which was discovered in Changbaishan Nature Reserve (China, Yanbian), and has faintly verrucose ellipsoid basidiospores similar to *C. clavocystidiatus*, but it can be readily distinguished by its furcate cheilocystidia and smooth to nearly smooth pileus surface [15]. *Crepidotus subfulviceps* (Murrill) Aime, Vila & P.-A. Moreau and *C. iqbalii* are close to the new species phylogenetically, but they are both stipitate *Crepidotus*, which can be readily distinguished [13,48].

## 4. Discussion

*Crepidotus* subg. *Dochmiopus* likely harbors a rich diversity of species in China. Despite numerous records of *C.* subg. *Dochmiopus* species in regional diversity studies in China, the documentation of new taxa remains limited [64,65,66,67,68]. For researchers new to *Crepidotus*, accurately identifying species is challenging due to the scarcity of molecular data and the limited number of available morphological characteristics, often leading to overlooked new taxa. Through thorough morphological analyses, the collation of key taxonomic features and the enrichment of molecular datasets can improve the accuracy of species identification, thereby increasing the likelihood of discovering new species and providing basic information to reveal the phylogenetic and taxonomic problems. 

Microscopic characters are essential for identifying species within *C.* subg. *Dochmiopus*, and requiring detailed morphological examinations to reveal more referable taxonomic characters for classification. This study utilized Clémençon’s description of pileipellis types, recognized patterns such as rectocutis, plagiotrichoderm and clavicutis based on hyphae arrangement and the orientation of terminal cells, thereby enhancing the referential value of pileipellis for interspecific identification, and considered that pileipellis and cheilocystidia patterns play a role in species recognition [69]. For species that are challenging to differentiate, we attempted to employ more characteristics for identification, including, habitat, stipe present or absence in mature specimens, and whether the lamellae edge is fimbriated. For example, *C. capitatocystidiatus* and *C. cesatii* were recognized by whether the lamellae edge is fimbriated and the pileus margin is striated [6]. To identify species more conveniently and precisely, we provide an identification key for elongated-spored species of *C.* subg. *Dochmiopus* known in China, 30 elongated-spored species of *C.* subg. *Dochmiopus*, including four new species described in this study. The use of DNA sequences, however, has simplified species recognition and improved accuracy. This study contributed by sequencing 15 specimens from four new species in both ITS and LSU regions, offering valuable molecular insights for future identification efforts. Thorough morphological research combined with molecular phylogenetics is crucial for the precise identification of *C.* subg. *Dochmiopus* species and the discovery of new taxa. 

The classification of *Crepidotus* needs to be re-evaluated, as some sections may be merged or new sections built in *Crepidotus.* Based on the phylogenetic tree constructed by combining ITS and LSU sequences, the phylogenetic framework of *Crepidotus* cannot support the classification of Hesler & Smith or Consiglio & Setti, which were proposed by morphological characteristics [5,6]. Both Hesler & Smith and Consiglio & Setti considered the distinction between *C.* subg. *Crepidotus* and *C.* subg. *Dochmiopus* to be the presence of clamp connections [5,6]. *Crepidotus epibryus*, *C. versutus*, and *C. cinnabarinus* were classified in subg. *Crepidotus* by absent clamp connections, but they are located in *C.* subg. *Dochmiopus* group in the phylogenetic tree. Additionally, the species of *C.* subg. *Dochmiopus* sect. *Autochthoni* possess smooth basidiospores. That hints smooth basidiospores and the absence of clamp connections may no longer be exclusive characteristics of *C.* subg. *Crepidotus.* Consiglio & Setti divided *C.* subg. *Crepidotus* into two sections and *C.* subg. *Dochmiopus* into three sections, including five series [6]. Previously, due to an incomplete comprehension of key taxonomic characteristics and the lack of phylogenetic studies, many sections were overlooked. Within this phylogenetic tree, the same sections were present in different clades. For example, Clade 2 and Clade 10 are both *C.* subg. *Dochmiopus* sect. *Autochthoni*. But morphologically, the cheilocystidia of Clade 2 species are lageniform, while the cheilocystidia of Clade 10 species are sinuously cylindrical to vine-like. There may be a new section among Clade 2 and Clade 10. However, relying on the current phylogenetic tree and morphological characteristics to build a new section is insufficient. More specimens, sequences and new gene segments are urgently needed to analyze and reveal the phylogenetic relationships within *C.* subg. *Dochmiopus* and *Crepidotus* to build a distinct classification based on morphology and phylogeny.
**Key to the elongated-spored species of *C.* subg. *Dochmiopus* known in China**1 Basidiospores smooth to nearly smooth21 Basidiospores ornamented102 Pileocystidia present*C. autochthonus*2 Pileocystidia absent33 Pileipellis gelatinous*C. betulae*3 Pileipellis non-gelatinous44 Cheilocystidia vine-shaped*C. trichocraspedotus*4 Cheilocystidia not vine-shaped55 Pileipellis hyphae clampless*C. albidus*5 Pileipellis hyphae clamped66 Lamellae edge not fimbriate76 Lamellae edge fimbriate87 Pileipellis a cutis*C. caspari*7 Pileipellis a tomentose*C. lamellomaculatus*8 Basidiospores width < 4.5 μm*C. albissimus*8 Basidiospores width ≥ 4.5 μm99 Pileipellis a cutis, pileus greyish white to silver-grey*C. occidentalis*9 Pileipellis a trichoderm, pileus white to light orange-yellow*C. ulmicola*10 Inhabit on living plants*C. herbaceus*10 Inhabit on rotten branches and woods1111 Pileus reddish*C. reticulatus*11 Pileus not reddish1212 Pleurocystidia present*C. luteicolor*12 Pleurocystidia absent1313 Pileocystidia present1413 Pileocystidia absent1514 Pileus buff yellow to apricot yellow*C. succineus*14 Pileus white*C. vulgaris*15 Basidiospores subglobose to ellipsoid, Q_m_ ≤ 1.601615 Basidiospores amygdaliform, oblong to cylindrical, Q_m_ > 1.60 2616 Pileus brown, at point of attachment smooth*C. payettensis*16 Pileus not brown, at point of attachment villous, fibrillose or tomentose1717 Pileus surface smooth1817 Pileus surface not smooth2018 Pileus translucent striate*C. capitatocystidiatus*18 Pileus not translucent striate1919 Lamellae edge not fimbriate, cheilocystidia apex bifurcate*C. furcaticystidiosus*19 Lamellae edge fimbriate, cheilocystidia apex branched or stag antlers*C. macedonicus*20 Pileus hygrophanous2120 Pileus non-hygrophanous2221 Cheilocystidia lageniform*C. lutescens*21 Cheilocystidia narrowly clavate to narrowly utriform*C. croceotinctus*22 Pileipellis with incrusted hyphae2322 Pileipellis without incrusted hyphae2423 Cheilocystidia clavate or ventricose, sessile*C. kauffmanii*23 Cheilocystidia clavate to narrowly clavate, pruinose stipe*C. clavocystidiatus*24 Pileus orange-yellow to brownish-yellow*C. yuanchui*24 Pileus white to cream2525 Basidiospores subglobose to broadly ellipsoid, Q ≤ 1.30*C. cesatii*25 Basidiospores ellipsoid to ovoid, Q > 1.30*C. subverrucisporus*26 Pileus light smoky or rusty brown*C. herbarum*26 Pileus white to light yellow or orange2727 Basidiospores length ≥ 8.0 μm*C. luteolus*27 Basidiospores length < 8.0 μm2828 Cheilocystidia with a long cylindrical protuberance at apex*C. tomentellus*28 Cheilocystidia without a long cylindrical protuberance at apex2929 Cheilocystidia filiform, narrowly lageniform, at times diverticular or knobby in the upper portion*C. neotrichocystis*29 Cheilocystidia clavate, utriform, strangulated, sometimes stag antlers*C. variabilis*

## Figures and Tables

**Table 1:** DNA sequences used in the phylogenetic analysis in this study.

Taxa	Voucher/Strain No.	Location	GenBank Sequence ID		Reference
			ITS	nLSU	
*C. affinis*	PDD:72848	New Zealand	KY827291	–	[34]
*C. alabamensis*	TBGT15610	India	MK459545	MK459543	[34]
*C. albolanatus*	PDD:72865	New Zealand	KY827292	–	[34]
*C. applanatus*	SLO 2534	Slovakia	OM832521	OM832556	[35]
*C. applanatus*	SLO 2539	Slovakia	OM832523	OM832558	[35]
*C. applanatus*	SLO 2551	Slovakia	OM832526	OM832560	[35]
*C. asiaticus*	TJB9995	Thailand	MF077337	MF077336	[8]
*C. asiaticus*	TJB10018	Thailand	MF077339	MF077338	[8]
*C. asiaticus*	FFAAS0336	China	MW580871	–	Direct Sub.
*C. asiaticus*	FFAAS0338	China	MW580872	–	Direct Sub.
*C. brunnescens*	MCA864	Not indicated	–	AF367936	Unpublished
*C. calolepis*	WU 28902	Hungary	KF879617	–	[36]
*C. calolepis*	iNaturalist 142786935	USA	OR824560	–	Unpublished
*C. calolepis*	314298	USA	MH188448	–	Direct Sub.
***C. capitatocystidiatus***	**FFAAS1310 Holotype**	**China**	**PQ061270**	**PQ061255**	**This study**
***C. capitatocystidiatus***	**FFAAS1311**	**China**	**PQ061271**	**PQ061256**	**This study**
***C. capitatocystidiatus***	**FFAAS1312**	**China**	**PQ061272**	**PQ061257**	**This study**
*C. caspari*	FFAAS0342	China	MZ401361	MW581521	[4]
*C. cinnabarinus*	MCA387	USA	–	AF205686	[37]
*C. circinatus*	MushroomObserver. org/307011	USA	MH087459	–	Direct Sub.
*C. circinatus*	iNAT:66988897	USA	OQ147188	–	Direct Sub.
*C. circinatus*	iNaturalist 141511315	USA	OR824682	–	Direct Sub.
***C. clavocystidiatus***	**FFAAS1316 Holotype**	**China**	**PQ061276**	**PQ061261**	**This study**
***C. clavocystidiatus***	**FFAAS1317**	**China**	**PQ061277**	**PQ061262**	**This study**
***C. clavocystidiatus***	**FFAAS1318**	**China**	**PQ061278**	**PQ061263**	**This study**
***C. clavocystidiatus***	**FFAAS1319**	**China**	**PQ061279**	**PQ061264**	**This study**
*C. croceotinctus*	iNat31834012	USA	MN498116	–	Direct Sub.
*C. croceotinctus*	S.D. Russell ONT iNaturalist # 126570732	USA	OP470431	–	Direct Sub.
*C. croceotinctus*	S.D. Russell ONT iNaturalist 131145003	USA	OP549098	–	Direct Sub.
*C. crocophyllus*	SLO 2433	Slovakia	OM832529	OM832562	[35]
*C. crocophyllus*	SLO 2588	Slovakia	OM832530	OM832563	[35]
*C. dentatus*	HMJAU37097	China	MH320736	–	[12]
*C. dentatus*	HMJAU37161	China	MH320737	–	[12]
*C. epibryus*	IBNR 1997/0948	Russia	–	AF367934	[38]
*C. epibryus*	NL-5379	Hungary	–	MK277884	[39]
*C. exiguus*	TBGT17176	India	–	MK567974	[11]
*C. flavobrunneus*	TBGT15841	India	–	MK567981	[11]
*C. fragilis*	MCA 904	USA	–	AF367931	[38]
*C. fraxinicola*	OKM26739	USA	–	AF205699	[37]
*C. fraxinicola*	OKM26748	USA	–	AF205697	[37]
*C. herbaceus*	HMJAU37009	China	MW080327	–	[20]
*C. herbaceus*	HMJAU37025	China	MW080326	–	[20]
*C. heterocystidiosus*	HMJAU37054	China	MF461342	–	[12]
*C. heterocystidiosus*	HMJAU37034	China	MF461344	–	[12]
*C. inhonestus*	MCA638	Not indicated	–	AF205704	[40]
*C. innuopurpureus*	MEL 2503290	Australia	NR_182391	MZ870346	[41]
*C. iqbalii*	MU248	Pakistan	OQ672617	–	Direct Sub.
*C. iqbalii*	LAH36654	Pakistan	MT973498	MW888515	[13]
*C. kangoliformis*	BAP 664	São Tomé and Príncipe	KX017199	–	[42]
*C. kauffmanii*	MIN-F-0905412	USA	–	MK277887	[39]
***C. lamellomaculatus***	**FFAAS1306**	**China**	**PQ061266**	**PQ061251**	**This study**
***C. lamellomaculatus***	**FFAAS1309**	**China**	**PQ061269**	**PQ061254**	**This study**
***C. lamellomaculatus***	**FFAAS1308**	**China**	**PQ061268**	**PQ061253**	**This study**
***C. lamellomaculatus***	**FFAAS1307**	**China**	**PQ061267**	**PQ061252**	**This study**
***C. lamellomaculatus***	**FFAAS1305 Holotype**	**China**	**PQ061265**	**PQ061250**	**This study**
*C. lanuginosus*	OKM27331	USA	–	AF367940	Unpublished
*C. lateralipes*	PDD:72508	New Zealand	KY827293	–	[34]
*C. lateralipes*	PDD:72571	New Zealand	KY827294	–	[34]
*C. lateralipes*	PDD:98270	New Zealand	KY827295	–	[34]
*C. luteolus*	16834	Italy	JF907963	–	[43]
*C. lutescens*	HMJAU 21976	China	KU762016	–	[7]
*C. lutescens*	HMJAU 37002	China	KU762017	–	[7]
*C. macedonicus*	PV773	Hungary	MH780921	–	[44]
*C. macedonicus*	DB3859	Hungary	MH780922	–	[44]
*C. macedonicus*	MB19102501	Italy	PP131267	PP125747	Direct Sub.
*C. malachioides*	SLO 2578	Slovakia	OM832538	OM832568	[35]
*C. malachioides*	SLO 2391	Slovakia	OM832536	OM832567	[35]
*C. malachius*	SLO 2541	Slovakia	OM832546	OM832575	[35]
*C. malachius*	SLO 2091	Slovakia	OM832541	OM832571	[35]
*C. malachius*	SLO 2530	Slovakia	OM832543	OM832573	[35]
*C. mollis*	OKM26279	USA	–	AF205677	[37]
*C. mollis*	PBM 1036 (WTU)	USA	–	DQ986293	[45]
*C. neotrichocystis*	CS1150	Malta	OL672745	OL672702	[46]
*C. nephrodes*	OKM25896	Not indicated	–	AF205693	[37]
*C. nephrodes*	MCA189	Not indicated	–	AF205670	[37]
*C. novae-zealandiae*	PDD:95850	New Zealand	HQ533046	–	Direct Sub.
*C. nyssicola*	S.D. Russell MycoMap # 7426	USA	MN906237	–	Direct Sub.
*C. nyssicola*	S.D. Russell MycoMap # 7399	USA	MN906236	–	Direct Sub.
*C. occidentalis*	MUOB:367585	USA	OK376745	–	Direct Sub.
*C. palodensis*	TBGT16716	India	MH844890	MH310743	[10]
*C. praecipuus*	PDD:72624	New Zealand	KY827312	–	[34]
*C. praecipuus*	PDD:72481	New Zealand	KY827311	–	[34]
*C. pseudomollis*	HMJAU37158	China	MH320739	–	[12]
*C. pseudomollis*	HMJAU37163	China	MH320740	–	[12]
*C. pseudomollis*	HMJAU37125	China	MH320738	–	[12]
*C. reticulatus*	HMJAU37089	China	MF461346	–	Direct Sub.
*C. roseus*	TBGT15507	India	MK567976	MK567977	[11]
*C. rufidulus*	PDD 98272	New Zealand	NR_159823	–	[34]
*C. rufofloccosus*	PDD 72601	New Zealand	NR_159822	–	[34]
*C. sphaerosporus*	11253	Italy	JF907960	–	Direct Sub.
*C. sphaerosporus*	HMAS 290002	China	MK966514	–	Direct Sub.
*C. stenocystis*	PRM911279	Czech Republic	MF621030	MF621024	[47]
*C. stenocystis*	SLO 481	Slovakia	OM832552	–	[35]
*C. stenocystis*	SLO 2557	Slovakia	OM832553	OM832581	[35]
*C. striatus*	HMJAU37087	China	MH320742	–	[12]
*C. striatus*	HMJAU37076	China	MH320741	–	[12]
*C. subfulviceps*	BCN SCM B-5144	Spain	–	FJ947116	[48]
*C. subfulviceps*	BCN SCM B-5138	Spain	–	FJ947117	[48]
*C. subverrucisporus*	MCA774	USA	–	AF367948	[19]
***C. succineus***	**FFAAS1313 Holotype**	**China**	**PQ061273**	**PQ061258**	**This study**
***C. succineus***	**FFAAS1314**	**China**	**PQ061274**	**PQ061259**	**This study**
***C. succineus***	**FFAAS1315**	**China**	**PQ061275**	**PQ061260**	**This study**
*C. tennesseensis*	TENN 29144	USA	FJ601806	–	Unpublished
*C. tennesseensis*	LRH29144	USA	NR_119720	GQ892981	[49]
*C. tobolensis*	TCSS UB RAS9477	Russia	MK522392	–	[50]
*C. tobolensis*	LE 287655	Russia	MK522393	MK560762	[50]
*C. tobolensis*	LE313671	Russia	OL739885	–	[51]
*C. tortus*	TBGT17194	India	MK462161	MK462162	[11]
*C. trichocraspedotus*	HMJAU37250	China	MH320744	–	[12]
*C. trichocraspedotus*	HMJAU37138	China	MH320743	–	[12]
*C. trulliformis*	PDD:98274	New Zealand	KY827298	–	[34]
*C. ulmicola*	HMJAU37011	China	KX456184	–	[20]
*C. ulmicola*	HMJAU37027	China	MW080328	–	[20]
*C. variabilis*	SLO 2021	Slovakia	MT055889	OM832585	[18]
*C. variabilis*	SLO 2018	Slovakia	MT055890	OM832583	[18]
*C. variabilis*	SLO 2423	Slovakia	MT055887	OM832584	[18]
*C. versutus*	MCA381	USA	–	AF205683	[37]
*C. versutus*	MCA250	USA	–	AF205695	[37]
*C. volubilis*	TBGT15648	India	MH845231	MH310742	[11]
*C. wasseri*	LE 287679	Russia	MW722981	MW723022	[52]
*C. wasseri*	MO500187	USA	OR203555	–	Direct Sub.
*C. yuanchui*	FFAAS0340	China	MZ401362	–	[4]
*C. yuanchui*	FFAAS0341	China	MZ401363	MW581519	[4]
*N. dominicanus*	MCVE 26928	Dominican Republic	JN033216	JN033217	[53]
*N. echinospermus*	AH45884	Brazil	KY468512	KY468511	Direct Sub.
*N. echinospermus*	MPM 2886	Brazil	–	JN033222	[53]

Remarks: New generated sequences are emphasized in bold; “–” show missing sequence.

## Data Availability

The original contributions presented in the study are included in the article, further inquiries can be directed to the corresponding author.
